# *ClC‐c* regulates the proliferation of intestinal stem cells via the EGFR signalling pathway in *Drosophila*

**DOI:** 10.1111/cpr.13173

**Published:** 2021-12-24

**Authors:** Jinping Huang, Xiao Sheng, Zhangpeng Zhuo, Danqing Xiao, Kun Wu, Gang Wan, Haiyang Chen

**Affiliations:** ^1^ Guangdong Province Key Laboratory of Pharmaceutical Functional Genes Key Laboratory of Gene Engineering of the Ministry of Education State Key Laboratory of Biocontrol School of Life Sciences Sun Yat‐sen University Guangzhou Guangdong China; ^2^ Laboratory of Metabolism and Aging Research National Clinical Research Center for Geriatrics West China Hospital Sichuan University Chengdu Sichuan China

**Keywords:** chloride channel, *Drosophila*, intestinal stem cells, stem cell proliferation, stem cell self‐renewal, tissue damage repair

## Abstract

**Objectives:**

Adult stem cells uphold a delicate balance between quiescent and active states, which is crucial for tissue homeostasis. Whereas many signalling pathways that regulate epithelial stem cells have been reported, many regulators remain unidentified.

**Materials and Methods:**

Flies were used to generate tissue‐specific gene knockdown and gene knockout. qRT‐PCR was used to assess the relative mRNA levels. Immunofluorescence was used to determine protein localization and expression patterns. Clonal analyses were used to observe the phenotype. RNA‐seq was used to screen downstream mechanisms.

**Results:**

Here, we report a member of the chloride channel family, *ClC*‐*c*, which is specifically expressed in *Drosophila* intestinal stem/progenitor cells and regulates intestinal stem cell (ISC) proliferation under physiological conditions and upon tissue damage. Mechanistically, we found that the ISC loss induced by the depletion of *ClC*‐*c* in intestinal stem/progenitor cells is due to inhibition of the EGFR signalling pathway.

**Conclusion:**

Our findings reveal an ISC‐specific function of *ClC*‐*c* in regulating stem cell maintenance and proliferation, thereby providing new insights into the functional links among the chloride channel family, ISC proliferation and tissue homeostasis.

## Graphical abstract

Our findings show that *ClC*‐*c* (the homologous gene of *ClC*‐*3* in *Drosophila*) is expressed in adult intestinal stem cells and is essential for proliferation during homeostasis and after tissue damage. This study demonstrates that *ClC*‐*c* regulates ISC proliferation through the EGFR signalling pathway.

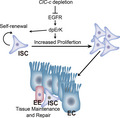

## 1 INTRODUCTION

In most adult somatic organs, resident adult stem cells, whose high proliferation and differentiation abilities compensate for cell loss, are responsible for both tissue homeostasis and organ functions throughout the lifespan of the organism. Compensation of cell loss is especially important in tissues with a high turnover rate, such as the intestinal epithelium.^1^ Intestinal stem cells (ISCs) undergo cell division and differentiation to rapidly replenish damaged cells, thereby maintaining intestine integrity during physiological turnover or in response to damage.^2^, ^3^ Thus, characterizing the regulators and signalling pathways that control stem cell activity and maintain the critical balance between cell generation and degeneration has been one of the main focuses of developmental biology and regenerative medicine. However, how cell‐fate transitions occur and how signalling pathways and transcriptional networks control these coordinated cellular changes remain largely unexplored.

**FIGURE 1:**
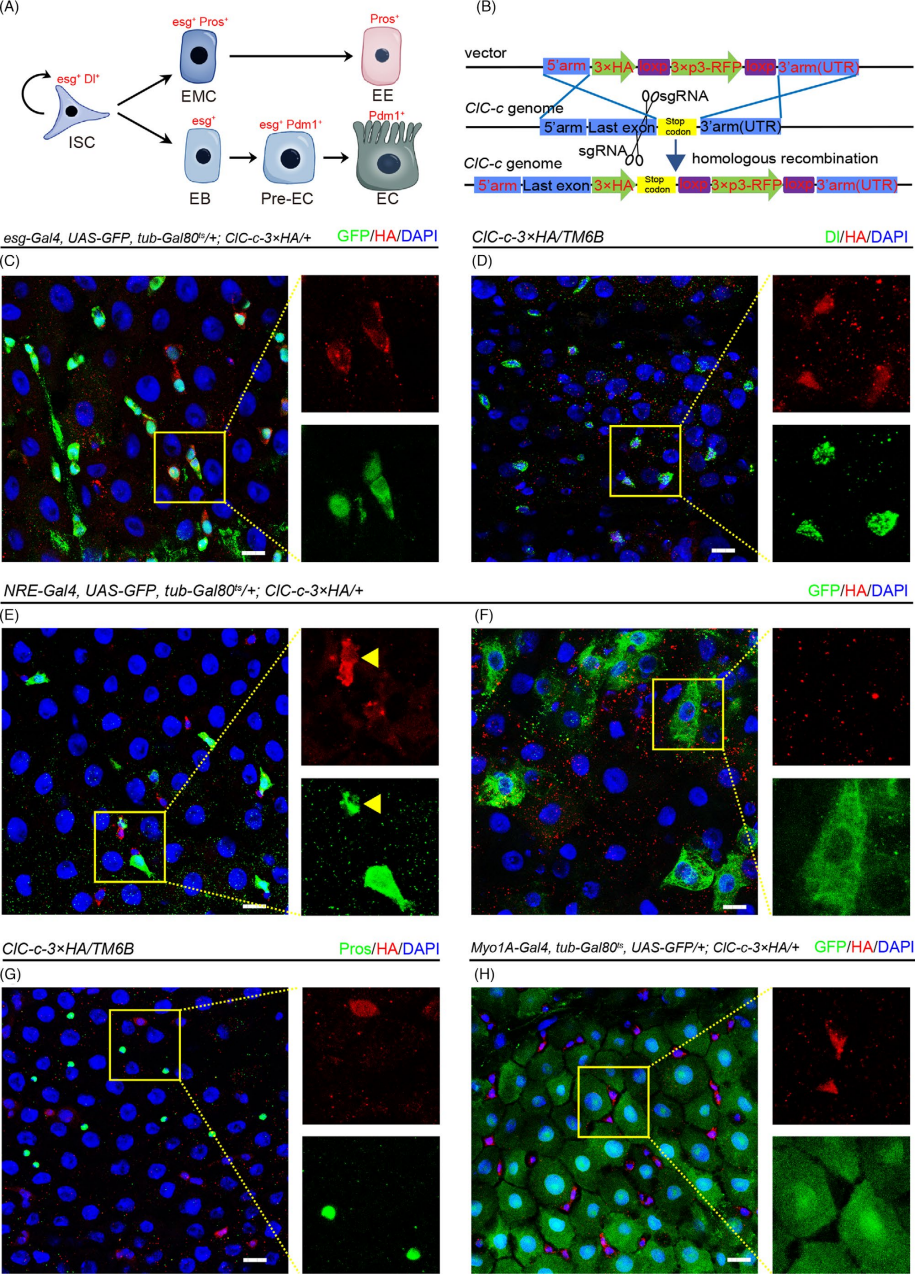
*ClC*‐*c* is specifically expressed in stem and progenitor cells in the intestinal epithelium of the adult *Drosophila*. (A) Schematic diagram of the cell types and markers in the *Drosophila* midgut. (B) Strategy of construction of *Drosophila* ClC‐c‐3 × HA knock‐in line. (C) Expression of ClC‐c‐3 × HA protein (red) in the *esg*‐*Gal4^ts^* > GFP^+^ cells (ISCs and EBs; co‐stained with GFP, green) in the R4 region of the *Drosophila* midgut. (D) Expression of ClC‐c‐3 × HA protein (red) in the ISCs (labelled by Delta staining, green) in the R4 region of the *Drosophila* midgut. (E and F) Expression of ClC‐c‐3 × HA protein (red) in the *NRE*‐*Gal4^ts^* > GFP^+^ cells (EBs; co‐stained with GFP, green) in the R4 region of the *Drosophila* midgut. Yellow arrowhead indicates the *NRE*‐*Gal4^ts^* > GFP^+^ cells with small nuclei (newly formed EBs). (G) Expression of ClC‐c‐3 × HA protein (red) in the EEs (labelled by Prospero staining, green) in the R4 region of the *Drosophila* midgut. (H) Expression of ClC‐c‐3 × HA protein (red) in the *Myo1A*‐*Gal4^ts^* > GFP^+^ cells (ECs; co‐stained with GFP, green) in the R4 region of the *Drosophila* midgut. EB, enteroblast; EC, enterocyte; ISC, intestinal stem cell. DAPI‐stained nuclei are shown in blue. Scale bar represents 10 μm

The *Drosophila melanogaster* intestine shares many similarities with its human counterpart and thus has emerged as a powerful system to study the role of intestinal stem cells in adult tissue homeostasis and regeneration because of its relatively simple and well‐characterized stem cell lineage and tractable genetic manipulation.^4^, ^5^ *Drosophila* ISCs are small diploid cells scattered along the basement membrane of the midgut epithelium and specifically express the transcription factor Escargot (Esg) and Notch ligand Delta (Dl).^2^, ^3^ The ISCs divide asymmetrically to generate new stem cells and either transient post‐mitotic progenitor cells named enteroblasts (EBs) or enteroendocrine mother cells (EMCs). EBs progressively differentiate into polyploid enterocytes (ECs), which are responsible for the absorption of nutrients in the midgut. The other type of differentiated cells in the *Drosophila* intestine is enteroendocrine cells (EEs). These cells emerge from EMCs, which express markers of both ISCs (Esg) and EEs (Prospero). A high level of Notch signalling drives ISCs to produce ECs, whereas a low level drives them to produce EEs (Figure 1A).^1^, ^4^, ^6^, ^7^, ^8^ Under homeostatic conditions, the ISCs in *Drosophila* midgut are largely quiescent. The number of ISCs and progenitor cells in the midgut is relatively small and remain stable. However, these ISCs promptly undergo proliferation and differentiation when the tissue is injured.^9^, ^10^ This cellular response is essential for maintaining the epithelial homeostasis since a failure to replace lost cells may compromise tissue function and overproduction of cells may lead to cancer. Under both homeostatic and stressed conditions, ISC divisions are modulated by numerous regulators, such as Sox21a, GATAe and Hairless,^11^, ^12^, ^13^ and signalling pathways, such as the EGFR, Wnt, mTOR and JAK/STAT pathways,^14^, ^15^, ^16^, ^17^, ^18^, ^19^, ^20^ to maintain the critical balance between cell generation and degeneration. However, how these signalling pathways are regulated and integrated by which is unclear.

Chloride channel‐3 (ClC‐3) is a member of the voltage‐dependent chloride channel ClC family, which functions in cell‐volume maintenance, cell excitability, lysosomal acidification, ion homeostasis and Cl‾ transport across the cell membrane.^21^, ^22^ Several diseases, including cystic fibrosis, neuronal ceroid lipofuscinosis, allergic rhinitis and myocardial ischemia,^23^, ^24^, ^25^, ^26^, ^27^ have been discovered to be associated with aberrant expression of ClC‐3. ClC‐3 has been shown to actively participate in various cancers, facilitating the aggressiveness and metastasis of various types of malignancies, including breast, cervical, prostate, and nasopharyngeal cancers and glioma tumours.^28^, ^29^, ^30^, ^31^, ^32^, ^33^ Additionally, ClC‐3 is expressed in human and mouse intestinal epithelium.^34^ Genetic deletion of ClC‐3 increases the susceptibility of mice to DSS‐ or TNBS‐induced experimental colitis and thus preventing the intestinal recovery.^34^ Although ClC‐3 has important roles in regulating the cell cycle, migration and apoptosis of tumour cells, its functions in intestinal stem cells are largely unknown.

This study found that *ClC*‐*c* (the homologous gene of *ClC‐3* in *Drosophila*) is specifically expressed in the ISCs and EBs of *Drosophila* midguts. Inhibition of *ClC*‐*c* expression in ISCs could inhibit their renewal. Additionally, *ClC*‐*c* was found to regulate ISC proliferation through the EGFR signalling pathway. This study not only uncovers a new function of the chloride channel gene *ClC*‐*c* in the modulation of ISC proliferation but also improves our understanding of ISC functions.

## 2 MATERIALS AND METHODS

### 2.1 *Drosophila* lines and husbandry

The following fly lines were obtained from the Bloomington *Drosophila* stock centre (BDSC): *w^1118^*, *UAS*‐*EGFR^CA^* (BDSC# 9533), *UAS*‐*stg*‐*HA* (BDSC# 56562), *UAS*‐*rpr* (BDSC# 5823), *UAS*‐*Rab5*‐*GFP* (BDSC# 43336), *UAS*‐*Rab7*‐*GFP* (BDSC# 42705), *UAS*‐*sSpi* (BDSC# 58436), *Df(3L)st*‐*e4* (BDSC# 1317) and *ClC*‐*c* RNAi (BDSC# 27034). *UAS*‐*Krn* (FlyORF # F002754) was obtained from the FlyORF.

The following fly lines were obtained from the Vienna *Drosophila* Resource Center (VDRC): *cic* RNAi (v103012), *cic* RNAi (v103805), *ClC*‐*c* RNAi (v6465) and *ClC*‐*c* RNAi (v6466).

The following fly lines were kindly provided as gifts: *esg*‐*GFP*, *esg*‐*Gal4*, *UAS*‐*lacZ*, *tub*‐*Gal4* and FTR2A lines by Dr. Allan Spradling; and *esg*‐*Gal4^ts^*, *MyolA*‐*Gal4^ts^*, *ISC*‐*Gal4^ts^* and *NRE*‐*Gal4^ts^* lines by Dr. Benjamin Ohlstein. All the *Drosophila* lines used in this study are listed in Table S1.

Flies were cultured in the standard medium (50 g cornmeal, 18.75 g yeast, 80 g sucrose, 20 g glucose, 5 g agar and 30 ml propionic acid per 1 L of media) at 25°C and with 65% humidity on a 12 h light/12 h dark cycle. Unless indicated otherwise, only females were used in this study.

### 2.2 Generation of the knockout, knock‐in and transgenic fly lines

To knock out *ClC*‐*c*, the following two sgRNAs located in the forepart of the CDS region of *ClC*‐*c* were designed using CRISPR Optimal Target Finder with the maximum stringency (http://tools.flycrispr.molbio.wisc.edu/targetFinder/). Each sgRNA was synthesized in vitro and then subcloned into the PMD18T vector to acquire the U6 promoter. Then, the U6 promoter and sgRNA were recombined into the attB vector. This sgRNA construct was injected into the fly line *y[1] w[1118]*; *VK00037* (BDSC# 9752) to generate *ClC*‐*c* sgRNA alleles. The *ClC*‐*c* sgRNA fly line was then crossed with the vas‐Cas9 fly line to obtain the mutant alleles. Two independent mutant alleles were confirmed by sequencing the *ClC*‐*c* locus and named *ClC*‐*c^d1^* and *ClC*‐*c^d2^*.

To generate *ClC*‐*c*‐*3*×*HA* knock‐in lines, two constructs were generated, one with two sgRNAs and the other with a homologous recombination sequence. The sgRNA construct was generated as mentioned above. To generate the homologous recombination construct, the 5ʹ homologous arm (~1 KB upstream sequence before the termination codon), 3 × HA and the 3ʹ homologous arm (~1 KB downstream sequence after the termination codon) were inserted into the PASK vector. All the final vectors were verified by sequencing and injected by Fungene Biotechnology (Beijing, China). The two sgRNAs were used to generate DNA double‐strand breaks. The 5ʹ and 3ʹ homologous arms were used for homologous recombination repair. 3 × P3‐RFP was used for screening. All the sgRNA sequence used are listed in Table S3.

To get UAS‐ClC‐c transgenic lines, the UAS‐ClC‐c expression vector was generated first. Full‐length cDNA (RT‐PCR from total mRNA of *w^1118^* flies) of *ClC*‐*c* was subcloned into the pEntry vector by using pEASY‐Uni Seamless Cloning and Assembly Kit (TransGen Biotech, CU101‐02) and then sub‐cloned into pTW vector by using LR recombination reaction. The primers are listed in Table S3.

### 2.3 Clonal analysis

*ClC*‐*c* mutant ‘Mosaic Analysis with a Repressible Cell Marker’ (MARCM) clones were generated from the *ClC*‐*c* mutant line via FLP/FRT‐mediated mitotic recombination. *FRT2A* was recombined with *ClC*‐*c^d1^* to generate the *FRT2A*, *ClC*‐*c*‐*null* mutant line, which was then crossed with the line *yw*, *hsFLP*, *tub*‐*Gal4*, *UAS*‐*GFP*/*FM7*; *tub*‐*Gal80*, *FRT2A*/*TM6B* to obtain *hsFLP*, *tub*‐*Gal4*, *UAS*‐*GFP*/+; *tub*‐*Gal80*, *FRT2A*/ *FRT2A*, *ClC*‐*c* flies. The flies were raised at 25°C, and 5‐day‐old adult flies post‐eclosion were subjected to 1‐h heat shock twice in a water bath at 37°C. Afterwards, they were kept at 25°C and then dissected and observed at the indicated days after clonal induction (ACI).

### 2.4 RNA‐sequencing (RNA‐seq) and data analysis

The detailed process was described previously.^35^ The *Drosophila* adult midguts (R1–R5) were first dissected in phosphate‐buffered saline (PBS) on ice. They were then immediately placed in a −80°C freezer. Total RNA was isolated using the isothiocyanate‐alcohol phenyl‐chloroform method. Whole RNA previous sequencing was carried out on the NovaSeq 6000 platform (Illumina) by Berry Genomics Corporation. The quality control was performed using FastQC (v0.11.8).

The read length of the raw RNA‐seq data was 150 bp. All the reads were aligned to the *Drosophila* reference genome (Ensembl BDGP6 release‐89). The aligned‐read sam files were then converted to bam files and sorted using SAMtools. DESeq2 (v1.22.2) was used to determine the gene expression profiles of samples. *P*‐value ≤ 0.05 following Benjamini and Hochberg correction for multiple hypothesis testing was considered to indicate differential gene expression.

### 2.5 Cell sorting and RT‐qPCR

One hundred to two hundred midguts were dissected in cold diethyl pyrocarbonate (DEPC)‐treated water PBS (0.1% final solution of DEPC is added to 1x PBS) and incubated with 0.1% trypsin for 1 h at 29°C, during which the sample was softly mixed every 15 min by pipetting and inverting several times. Dissociated samples were collected after centrifugating at 4°C, 400× *g* for 20 min and resuspended in DEPC‐PBS. The suspension was filtered with 40‐μm filters. The filtered cells are then sorted using a FACS Aria II sorter (BD Biosciences) and collected into 0.5 ml DEPC‐PBS. GFP^+^ cells in the midgut of fly strains carrying *esg*‐*Gal4^ts^* ^^> *UAS*‐*GFP* fluorescent markers were used to sort intestinal progenitor cell population, and the midguts of *w^1118^* flies were set as fluorescence gate.

Total gut RNA was extracted from dissected midguts by using the Trizol reagent (Invitrogen). This RNA (1 μg) was used to generate cDNA via reverse transcription, and the cDNA was subjected to quantitative polymerase chain reaction (qPCR) in a QuantStudio 5 System (Thermo Fisher Scientific). The 2^−ΔΔCT^ method was used to calculate the expression values. The relative expression was normalized to that of *Rp49*. All the primers used are listed in Table S3.

### 2.6 Immunofluorescence microscopy

Adult midguts were dissected in PBS and then fixed with 4% Paraformaldehyde for 2 h, followed by washing three times (10 min each) with PBS containing 0.1% Tween‐20 (PBST). The midguts were then blocked in 0.1% BSA for 30 min at room temperature, followed by washing with PBST, and then incubated overnight at 4°C with primary antibodies diluted in PBST. After washing three times with PBST, the tissues were incubated with secondary antibodies and DAPI for 2 h at room temperature, followed by the same washing steps above. The sources and dilutions of the primary and secondary antibodies used are listed in Table S2.

Leica TCS‐SP8 confocal microscope was used to acquire all the immunofluorescence images. The Leica Application Suite X (LAS X), Adobe Photoshop cc2020 and Adobe Illustrator cc2020 were used to assemble the images.

### 2.7 Bleomycin (BLM), dextran sodium sulphate (DSS) and paraquat (PQ) treatment

A chromatography paper was cut into 4 × 6 cm strips and saturated with 25 µg/ml BLM (Aladdin, B107423), 5% (wt/vol) DSS (MP Biomedicals, CAS: 9011–18–1, 36–50 KD) or 5 mM PQ (Aladdin, M106761) dissolved in 5% (wt/vol) sucrose. After being starved in empty vials for 1 h, flies were transferred into vials with the BLM, DSS or PQ solution–saturated chromatography paper with 5% sucrose. After 24 h (for BLM or PQ) or 48 h (for DSS) of treatment, the flies were transferred to the standard fly food, with the daily renewal of the food. Sucrose‐only (5%, wt/vol) was used as a control.

### 2.8 TUNEL assay

Adult midguts were dissected in PBS and fixed with 4% paraformaldehyde for 2 h, followed by washing with PBST. Apoptosis was assessed using the Apoptag Kit (Millipore) according to the manufacturer's instructions.

### 2.9 EdU incorporation assay

For EdU labelling, a chromatography paper was cut into 4 × 6 cm strips and saturated with 5% sucrose and 100 µM EdU (Baseclick). After being starved in empty vials for 1 h, flies were transferred into vials with the EdU solution–saturated chromatography paper for 24 h. The guts of the flies were then dissected in PBS and fixed with 4% paraformaldehyde for 2 h. Edu incorporation was evaluated following the manufacturer's instructions. The immunostaining procedure has previously been described.

### 2.10 Detection of reactive oxygen species (ROS) by using dihydroethidium (DHE)

Adult midguts were dissected in PBS and then incubated in 30 μM DHE (Invitrogen) for 5 min in the dark at room temperature. The guts were then washed twice with PBS, mounted and immediately examined using a confocal microscope.

### 2.11 Assessment of necrosis by using propidium iodide

Adult midguts were dissected in PBS and then stained with 1.5 μM propidium iodide (PI; Invitrogen) at room temperature for 15 min. The guts were then fixed with 4% formaldehyde for 20 min, washed three times with PBST, rinsed twice with PBS, mounted in Vectashield with DAPI and examined using a confocal microscope.

### 2.12 Fluorescence intensity statistics

Immunofluorescence images were analysed via confocal microscopy, and the fluorescence intensity statistics in the region of interest (ROI) were calculated using ImageJ. The detailed process was described previously.^35^

Mean fluorescence intensity = Integrated density of the background region (or cell)/Area of the background region (or cell)

Relative mean fluorescence intensity = Mean fluorescence intensity of the cell−Mean fluorescence intensity of the background region

Relative fluorescence intensity = Integrated density of the cell−Mean fluorescence intensity of the background region × Area of the cell

### 2.13 Quantification and statistical analysis

For all the experiments, the data were processed using GraphPad Prism and presented as average ± SD. Statistical significance was determined using the unpaired two‐tailed Student's *t*‐test unless otherwise stated in the Figure legends. For all the tests, *p* < 0.05 was considered to indicate statistical significance.

## 3 RESULTS

### 3.1 *ClC*‐*c* is specifically expressed in the stem and progenitor cells of the adult *Drosophila* intestinal epithelium

To investigate the possible role of *ClC*‐*c* in the regulation of intestinal stem cells, a *ClC*‐*c*‐*3*×*HA* knock‐in fly line was generated (Figure 1B). We used the conditional temperature‐sensitive driver *esg*‐*Gal4* (*esg*‐*Gal4^ts^*) to express the dsRNA construct (BDSC # 27034) against *ClC*‐*c* and observed that the HA signal was strongly reduced (Figure S1A–C). We detected ClC‐c expression in R1–R5 regions of the adult *Drosophila* midgut (Figure S1D). Using specific cell markers to distinguish the cell‐specific expression pattern of the endogenous ClC‐c protein, we found that *ClC*‐*c* is specifically expressed in *esg*‐positive cells (Figure 1C). By using e*sg*‐*Gal4*, *UAS*‐*GFP*; *Gal80^ts^* and *NRE*‐*Gal80 (ISC*‐*Gal4^ts^)* flies, we found that *ClC*‐*c* is expressed in ISCs (Figure S1E). Additionally, co‐staining the *ClC*‐*c*‐*3* × *HA* midguts for the Dl protein and HA tag confirmed that ClC‐c is expressed in ISCs (Figure 1D). Next, we found that ClC‐c is expressed in some newly formed EBs with small nuclei (Figure 1E, yellow arrowhead), whereas no ClC‐c expression was detected in mature EBs with large nuclei (Figure 1F). These results imply that the expression of ClC‐c is gradually lost during the differentiation from EBs to ECs. As predicted, no ClC‐c expression was detected in mature ECs (Figure 1H) or EEs (Figure 1G). Taken together, these results indicate that *ClC*‐*c* is specifically expressed in the stem/progenitor cells of the *Drosophila* intestinal epithelium.

### 3.2 *ClC*‐*c* is required for ISC proliferation in the *Drosophila* midguts

**FIGURE 2:**
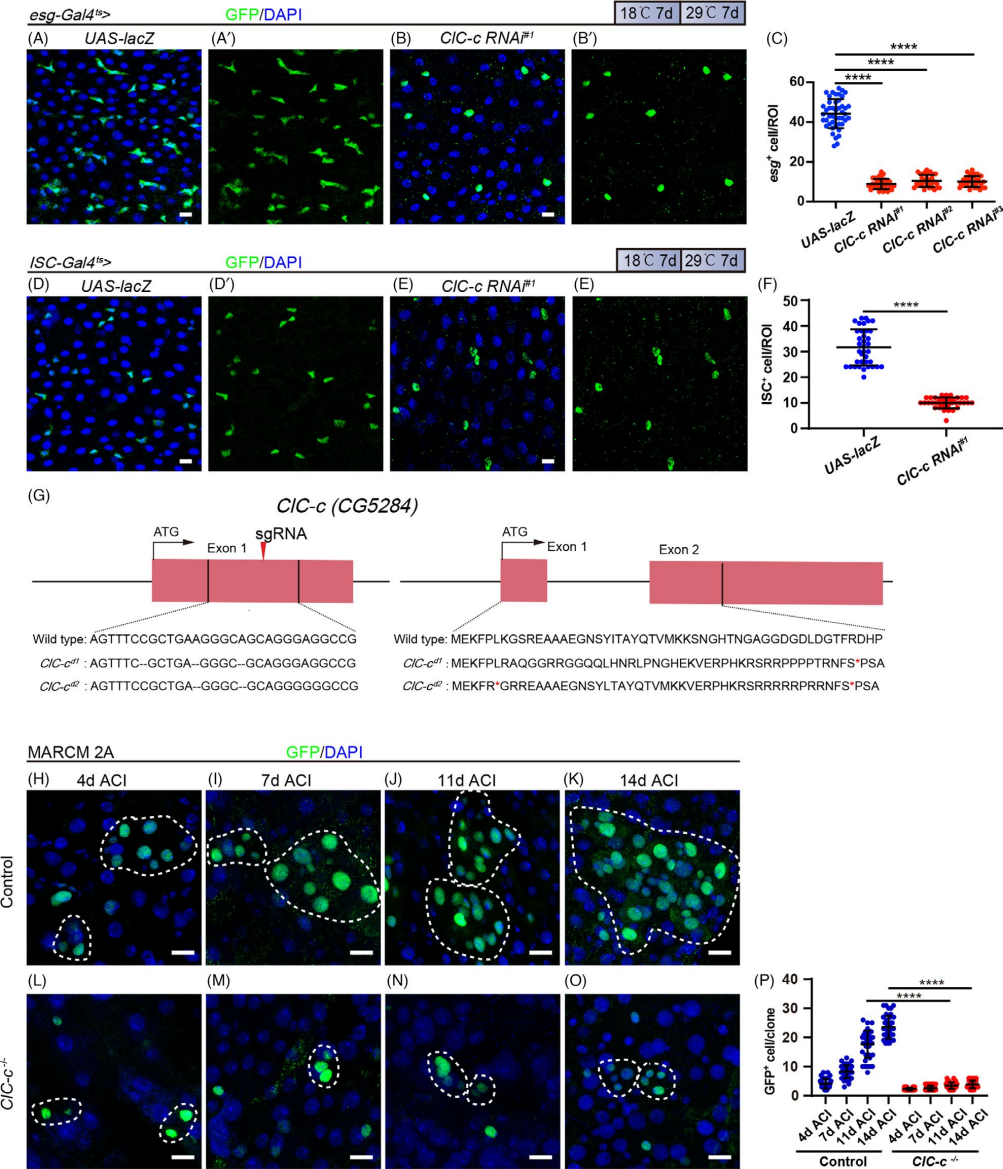
*ClC*‐*c* is required for ISC proliferation in the *Drosophila* midgut. (A and B) Immunofluorescence images from the R5 region of the midgut of the control flies (A, *esg*‐*Gal4^ts^*‐driven *UAS*‐*lacZ*) or *ClC*‐*c*‐depleted flies (B, *esg*‐*Gal4^ts^*‐driven *ClC*‐*c RNAi^#1^*, BDSC# 27034). (C) Quantification of the number of the *esg* ^+^ cells in the midguts of the control and *ClC*‐*c RNAi* groups. ROI: 2 × 10^4^ µm² area from the R5 region of the *Drosophila* midgut. Gut *n* ≥ 10; ROI *n* ≥ 30. (D and E) Immunofluorescence images from the R5 region of the midgut of the control flies (D, *ISC*‐*Gal4^ts^*‐driven *UAS*‐*lacZ*) or *ClC*‐*c*‐depleted flies (E, *ISC*‐*Gal4^ts^*‐driven *ClC*‐*c RNAi^#1^*, BDSC# 27034). GFP (green) identifies ISCs. (F) Quantification of the number of the GFP^+^ cells in (D) and (E). ROI: 2 × 10^4^ µm² area from the R5 region of the *Drosophila* midgut. Gut *n* ≥ 10; ROI *n* ≥ 30. (G) Schematic summary of the *ClC*‐*c* mutant alleles generated using CRISPR‐Cas9. (H–O) Immunofluorescence analyses of the control (*FRT2A*, H–K) and *ClC*‐*c^d1^* mutant (L‐O) MARCM clones (green) in the midguts 4, 7, 11 and 14 d after clonal induction (ACI). (P) Quantification of the cell number per MARCM clone in H‐O. Each dot corresponds to one clone. Gut *n* ≥ 10; clone *n* ≥ 30. ISC, intestinal stem cell; MARCM, mosaic analysis with a repressible cell marker; ROI, region of interest. DAPI‐stained nuclei are shown in blue. Scale bar represents 10 μm. Error bars represent SDs. Student's *t*‐test, ****p < 0.0001

Since the expression of *ClC*‐*c* is uniquely restricted to ISCs and some newly formed progenitor cells, we first depleted *ClC*‐*c* in ISCs and EBs via *esg*‐*Gal4^ts^* driven RNA interference (RNAi) lines *ClC*‐*c* RNAi^#1^ (BDSC# 27034), *ClC*‐*c* RNAi^#2^ (VDRC v6465) and *ClC*‐*c* RNAi^#3^ (VDRC v6466). The *ClC*‐*c* mRNA levels in these three RNAi lines were measured via RT‐qPCR (Figure S2A). Knocking down *ClC*‐*c* in the *esg* ^+^ cells for 7 days at 29°C significantly decreased the number of *esg* ^+^ cells (Figure 2A–C, Figure S2C–E). To further determine in which cell types *ClC*‐*c* exerts its functions, we knocked down *ClC*‐*c* in either ISCs or EBs by using the following cell‐specific Gal4 drivers: *ISC*‐*Gal4^ts^* for ISC knockdown; and *NRE*‐*Gal4*, *Gal80^ts^ (NRE*‐*Gal4^ts^)* for EB knockdown. We found that depletion of *ClC*‐*c* in ISCs decreases the number of *ISC* ^+^ cells (Figure 2D–F), whereas no effects on the number of EBs in depletion of *ClC*‐*c* in *NRE* ^+^ cells (Figure S2F–H). Additionally, depletion of *ClC*‐*c* in EBs did not affect the number of *esg* ^+^ cells (Figure S2I–K). We also tested the effects of overexpression of *ClC*‐*c* on ISCs. Overexpression of *ClC*‐*c* did not significantly promote ISC proliferation (Figure S2L‐N). These results suggest that *ClC*‐*c* might act cell‐autonomously in ISCs to regulate ISC proliferation.

Next, *ClC*‐*c* mutant alleles were generated using CRISPR/Cas9 to validate the *ClC*‐*c* RNAi phenotypes. We designed a sgRNA targeting the first exon of *ClC*‐*c* and isolated two independent lines, each carrying one of the mutant alleles *ClC*‐*c^d1^* and *ClC*‐*c^d2^*. By sequencing the *ClC*‐*c* locus, both lines were confirmed to be mutant as many base deletions occurred after position c.392 in exon 1, thus introducing a premature termination codon into this exon in both lines (Figure 2G). No progeny was obtained when these lines were crossed with the corresponding deficiency line Df (3L) st‐e4, in which the entire ClC‐c locus is deleted (Figure S2B). These results suggest that *ClC*‐*c^d1^* and *ClC*‐*c^d2^* are null or loss‐of‐function alleles.

To observe the phenotypes of the *ClC*‐*c* mutants, we used the MARCM system in which both the control and *ClC*‐*c* mutant clones were marked by GFP expression. The GFP‐marked clones were analysed at the indicated time points after ACI. The control clones showed normal growth at days 4, 7, 11 and 14 (Figure 2H–K,P, and Figure S2O), whereas the growth of the *ClC*‐*c* mutant clones was inhibited (Figure 2L–P). This result suggests that the *ClC*‐*c* mutant clones have a restricted capacity to divide and generate progeny.

Taken together, these results strongly suggest a cell‐autonomous role for *ClC*‐*c* in regulating ISC proliferation.

### 3.3 *ClC*‐*c* is required for midgut regeneration

**FIGURE 3:**
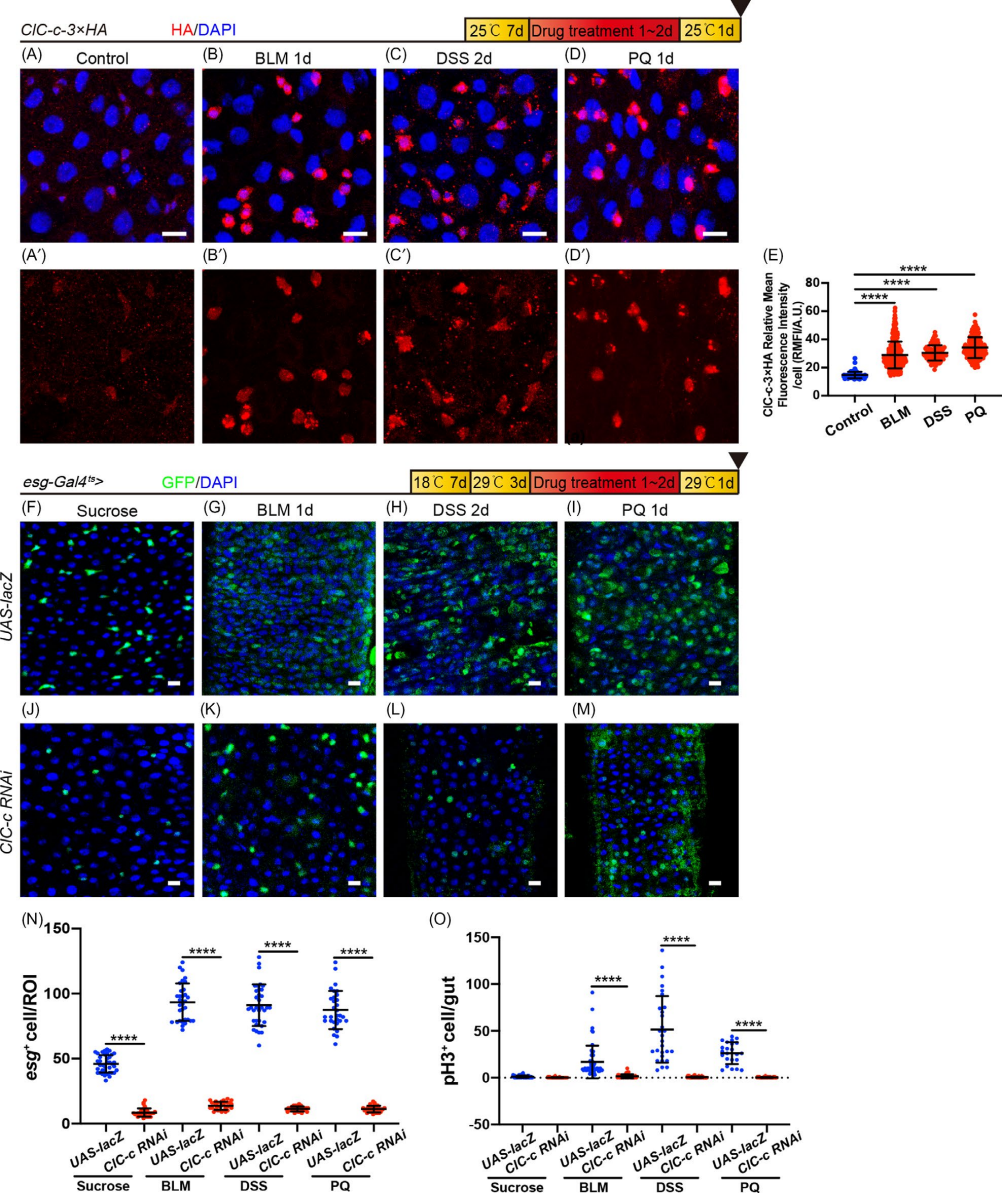
*ClC*‐*c* is required for midgut regeneration. (A–D) Immunofluorescence images from the R5 region of the midgut, showing the expression of ClC‐c‐3 × HA protein (red) upon Sucrose (A), BLM (B), DSS (C) or PQ (D) treatment. (E) Quantification of the fluorescence intensities of ClC‐c‐3 × HA in the midguts in (A–D). Each dot corresponds to one cell. Gut *n* ≥ 5; cell *n* ≥ 90. (F–I) Immunofluorescence images from the R5 region of the midgut of the control flies (*esg*‐*Gal4^ts^*‐driven *UAS*‐*lacZ*) treated with sucrose (F), BLM (G), DSS (H) or PQ (I). (J–M) Immunofluorescence images from the R5 region of the midgut of *ClC*‐*c*‐depleted flies (*esg*‐*Gal4^ts^*‐driven *ClC*‐*c RNAi^#1^*, BDSC# 27034) treated with sucrose (J), BLM (K), DSS (L) or PQ (M). (N) Quantification of the number of the GFP^+^ cells in the midguts in (F–M). ROI: 2 × 10^4^ µm² area from the R5 region of the midgut. Gut *n* ≥ 10; ROI *n* ≥ 30. (O) Quantification of the number of the pH3^+^ cells in the midguts in F–M. BLM, Bleomycin; DAPI labels the nucleus (blue); DSS, Dextran Sodium Sulphate; PQ, Paraquat. ROI, region of interest. Scale bar represents 10 μm. Error bars represent SDs. Student's *t*‐test, ****p < 0.0001. Gut *n* ≥ 20

Intestinal stem cells confer a high regenerative capacity to the intestinal epithelium.^36^ They undergo rapid cell division and differentiation to replenish damaged cells, thereby maintaining tissue integrity and the proper number of differentiated cells under the homeostatic conditions or in response to stress.^37^ *ClC*‐*c* has been shown to be involved in ISC proliferation during homeostasis. Therefore, we utilized the *ClC*‐*c*‐*3*×*HA* reporter line to visualize the expression level of ClC‐c in *esg* ^+^ cells in injured midguts. The abundance of ClC‐c considerably increased in BLM (causes DNA breaks and genomic instability)‐, DSS (a model for experimental inflammatory bowel disease)‐ or PQ (induces oxidative stress)‐injured midgut epithelium (Figure 3A–E). Treatment of the *Drosophila* intestinal tract with BLM, DSS or PQ caused intestinal damage and induced ISC hyperproliferation as indicated by the increase in the numbers of *esg* ^+^ and pH3^+^ cells (Figure 3F–I,N,O). However, the numbers of *esg* ^+^ and pH3^+^ cells in the intestines of the flies with depleted *ClC*‐*c* did not increase when these flies were subjected to these injuries (Figure 3J–O). These results suggest that *ClC*‐*c* is essential for inducing ISC proliferation in response to damage to the intestinal epithelium.

### 3.4 Reducing *ClC*‐*c* inhibits the proliferation of progenitor cells, but does not lead to apoptosis or necrosis

**FIGURE 4:**
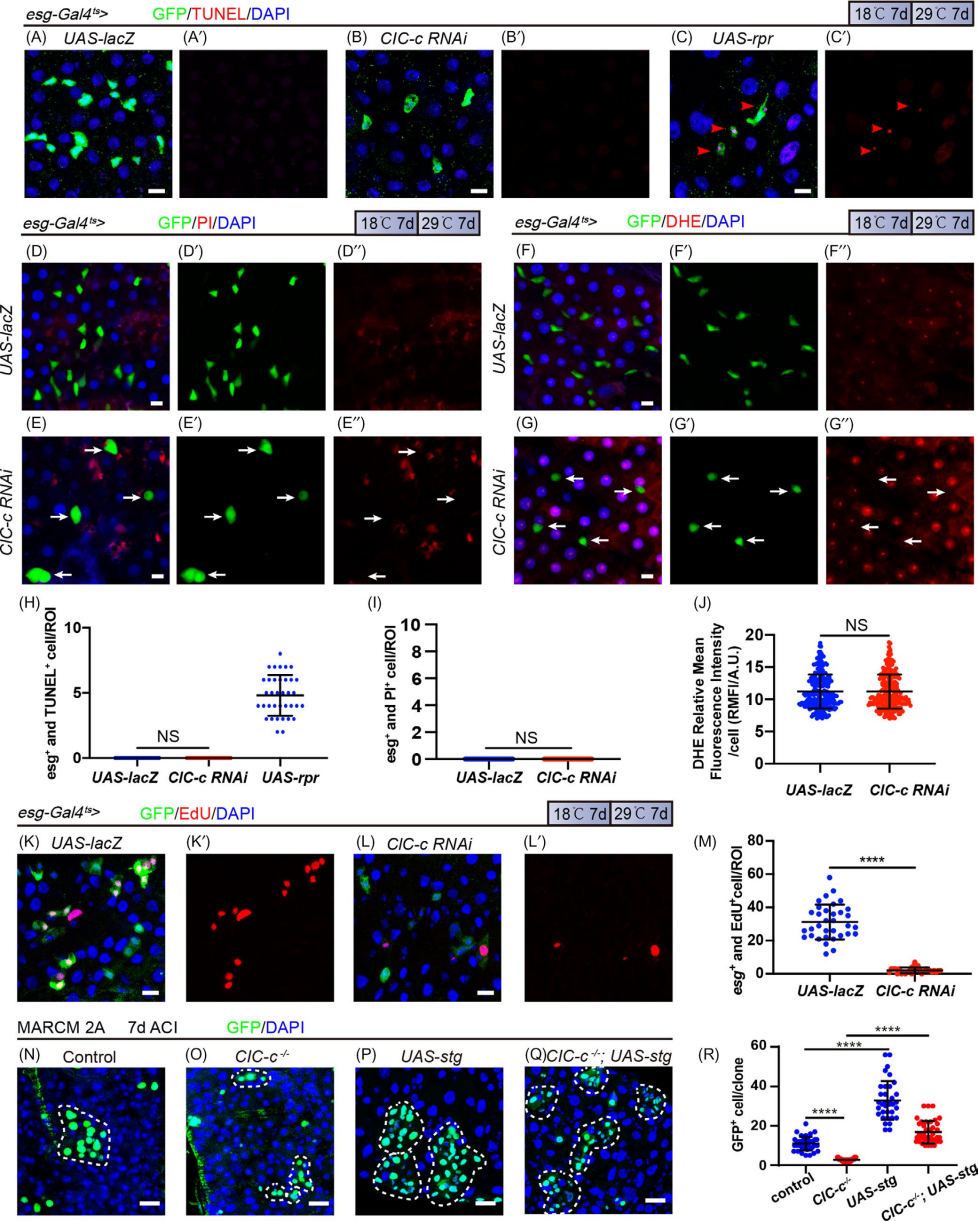
Reducing *ClC*‐*c* leads to a loss of progenitor cells due to decreased proliferation but not increased apoptosis or necrosis. (A–C) Immunofluorescence images from the R5 region of the midgut of the negative‐control flies (A, *esg*‐*Gal4^ts^*‐driven *UAS*‐*lacZ*), *ClC*‐*c*‐depleted flies (B, *esg*‐*Gal4^ts^*‐driven *ClC*‐*c RNAi*) and positive‐control flies (C, *esg*‐*Gal4^ts^*‐driven *UAS*‐*rpr*) with TUNEL treatment. (D and E) Immunofluorescence images from the R5 region of the midgut of the control flies (D, *esg*‐*Gal4^ts^*‐driven *UAS*‐*lacZ*) and *ClC*‐*c*‐depleted flies (E, *esg*‐*Gal4^ts^*‐driven *ClC*‐*c RNAi*) with PI treatment. White arrows indicate *esg* ^+^ cells. (F and G) Immunofluorescence images from the R5 region of the midgut of the control flies (F, *esg*‐*Gal4^ts^*‐driven *UAS*‐*lacZ*) and *ClC*‐*c*‐depleted flies (G, *esg*‐*Gal4^ts^*‐driven *ClC*‐*c RNAi*) with DHE treatment. White arrows indicate *esg* ^+^ cells. (H) Quantification of the number of the GFP^+^ and TUNEL^+^ cells in the midgut in (A), (B) or (C). ROI: 2 × 10^4^ µm² area from the R5 region of the *Drosophila* midgut. Gut *n* ≥ 10; ROI *n* ≥ 30. (I) Quantification of the number of the GFP^+^ and PI^+^ cells in the midgut in (D) or (E). ROI: 2 × 10^4^ µm² area from the R5 region of the *Drosophila* midgut. Gut *n* ≥ 10; ROI *n* ≥ 30. (J) Quantification of the number of the GFP^+^ and DHE^+^ cells in the midgut in (F) or (G). ROI: 2 × 10^4^ µm² area from the R5 region of the *Drosophila* midgut. Gut *n* ≥ 10; cell *n* ≥ 200. (K and L) Immunofluorescence images from the R5 region of the midgut of the control flies (K, *esg*‐*Gal4^ts^*‐driven *UAS*‐*lacZ*) and *ClC*‐*c*‐depleted flies (L, *esg*‐*Gal4^ts^*‐driven *ClC*‐*c RNAi*) with EdU treatment. (M) Quantification of the number of the GFP^+^ and EdU^+^ cells in the midgut in (K) or (L). ROI: 2 × 10^4^ µm² area from the R5 region of the *Drosophila* midgut. Gut *n* ≥ 10; ROI *n* ≥ 30. (N–Q) Immunofluorescence analyses of the control (N), *ClC*‐*c^d1^*‐null (O), *stg*‐overexpressing (P) and *stg*‐overexpressing *ClC*‐*c^d1^*‐null (Q) MARCM clones (green) in the midgut 7 days after clonal induction (ACI). (R) Quantification of the cell number per MARCM clone in (N–Q). Each dot corresponds to one clone. Gut *n* ≥ 10; clone *n* ≥ 30. DHE, dihydroethidium; MARCM, mosaic analysis with a repressible cell marker; PI, propidium iodide; ROI, region of interest. DAPI‐stained nuclei are shown in blue. Scale bar represents 10 μm in (A–G, K, L) and 25 μm in N–Q. Error bars represent SDs. Student's *t*‐test, ****p < 0.0001. NS, non‐significant (*p* > 0.05)

Knocking down *ClC*‐*c* in the *esg* ^+^ progenitor cells and clones decreased the number of *esg* ^+^ cells and resulted in small clones respectively. Apoptosis is a common reason for cell loss. To assess the possibility that the *esg* ^+^ cells undergo apoptosis upon knocking down *ClC*‐*c*, the TUNEL assay was performed to detect apoptotic signals after 7 days of *ClC*‐*c* RNAi. We did not observe a significant increase in the number of apoptotic *esg* ^+^ cells, compared with the number in the control flies (Figure 4A,B,H). Forced expression of *rpr* (an inducer of apoptosis) in *esg* ^+^ cells was used as a positive control (Figure 4C,H). These results indicate that the cell loss caused by knocking down *ClC*‐*c* in *esg* ^+^ cells is not due to an increase in apoptosis.

Another possibility of cell death is necrosis. Therefore, we investigated whether knocking down *ClC*‐*c* causes necrosis in ISCs. Necrosis is characterized by early plasma‐membrane rupture and accumulation of ROS, which can be assessed through PI staining and DHE staining respectively.^38^ We detected no increase in PI^+^ or DHE^+^ signals after *ClC*‐*c* depletion, compared with the signals in the control (Figure 4D–G,I,J).

Taken together, these results suggest that knocking down *ClC*‐*c* does not induce apoptosis or necrosis in ISCs.

During homeostasis, the stability of a stem‐cell pool requires continuous self‐renewal of the stem cells. Inhibition of their proliferation results in the depletion of the stem‐cell pool and loss of stem cells. To determine whether *ClC*‐*c* is required for the proliferation of ISCs, we performed EdU incorporation assay (a method to detect cell cycle) in *esg*‐*gal4^ts^* > GFP flies after *ClC*‐*c* RNAi and quantified the number of EdU‐positive cells per midgut. After 7 d of RNAi, the number of EdU^+^ cells was lower in these flies than in the controls (Figure 4K–M). Furthermore, forced expression of the cell cycle regulator *stg* rescued the cell loss phenotype induced by *ClC*‐*c* depletion (Figure 4N–R and Figure S3A–E). These results suggest that *ClC*‐*c* is required in maintaining ISC proliferation and the depletion of *ClC*‐*c* suppressed ISC proliferation by inhibiting the cell cycle.

The differentiation of stem cell is another possible cause of the decline in the number of stem cells. We thus investigated the effect of *ClC*‐*c* depletion on ISC differentiation. The experimental results showed that *ClC*‐*c* mutant did not inhibit the formation of large nuclear ECs and pros^+^ EEs (Figure S3F,G). Moreover, statistical data showed that the proportion of EC cells in the *ClC*‐*c* mutant clone was higher than that in the control group (Figure S3H).

Therefore, we believed that the decrease in the number of stem cells caused by the loss of *ClC*‐*c* is due to the other fact that ISC could not maintain stemness thus undergoing differentiation.

### 3.5 Depletion of *ClC‐c* downregulates the members of the EGFR signalling in the *Drosophila* midgut

**FIGURE 5:**
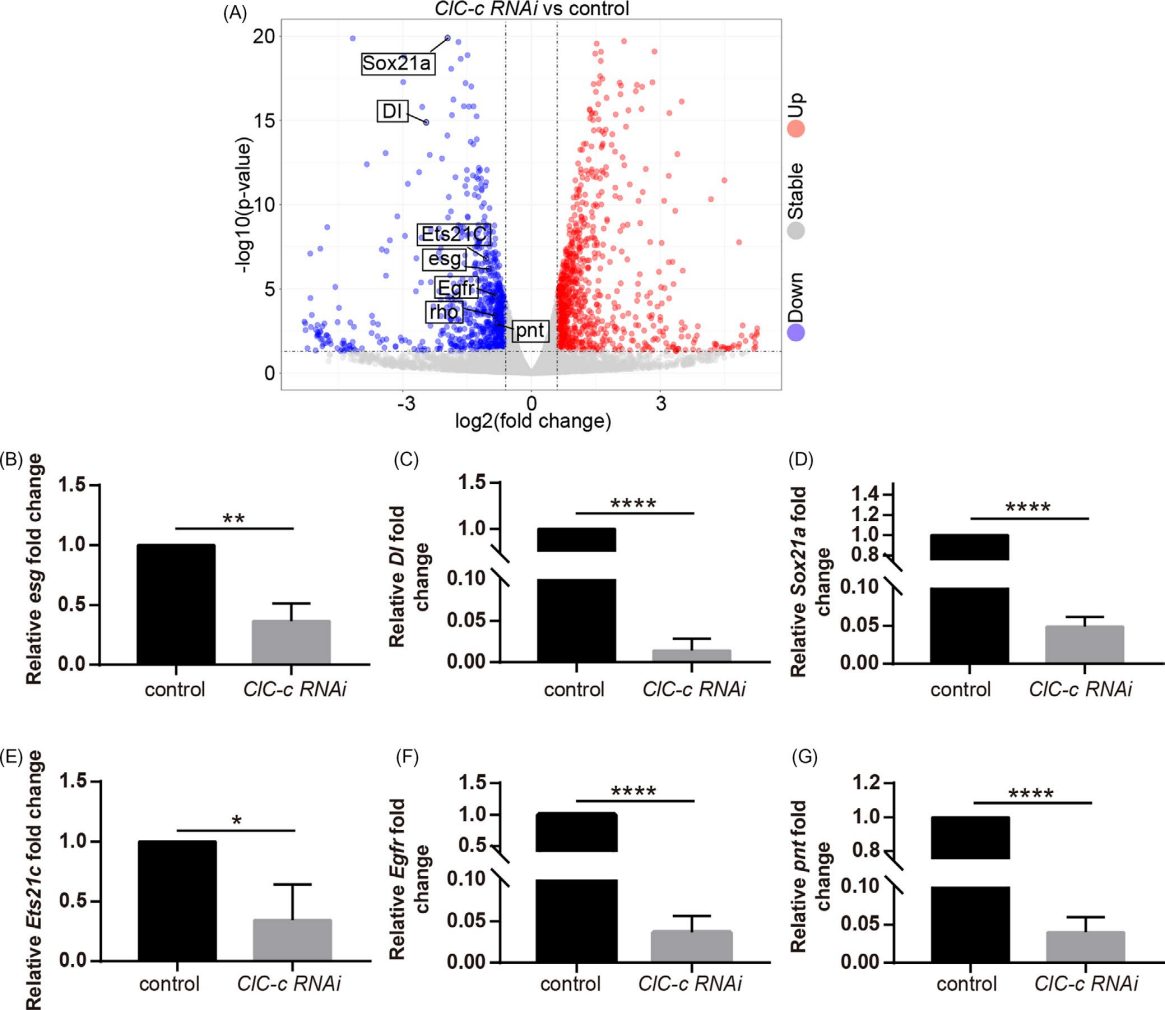
Depletion of *ClC*‐*c* downregulates genes related to the EGFR signalling in the *Drosophila* midgut. (A) Volcano plots of differentially expressed genes between the *ClC*‐*c*‐depleted *Drosophila* (*esg*‐*Gal4^ts^*‐driven *ClC*‐*c RNAi*) and control (*esg*‐*Gal4^ts^*‐driven *UAS*‐*lacZ*) midguts. (B–G) Relative mRNA levels of the genes related to the EGFR signalling in the *ClC*‐*c*‐depleted *Drosophila* (*esg*‐*Gal4^ts^*‐driven *ClC*‐*c RNAi*) and control (*esg*‐*Gal4^ts^*‐driven *UAS*‐*lacZ*) midguts. Error bars indicate the SD of three independent experiments. Student's *t*‐test, *p < 0.05, **p < 0.01, ****p < 0.0001

To identify the mechanism whereby *ClC*‐*c* regulates ISC proliferation, RNA‐seq was performed on dissected midguts of the flies with *ClC*‐*c* depleted in *esg* ^+^ cells (*esg*‐*Gal4^ts^* > *ClC*‐*c* RNAi) and control flies (*esg*‐*Gal4^ts^* > *UAS*‐*lacZ*). The expression levels of *esg* and *DI* were significantly lower in the midguts with *ClC*‐*c*‐depleted *esg* ^+^ cells than in the midguts of the control group (Figure 5B,C). This observation is consistent with the role of *ClC*‐*c* in maintaining the stemness of ISCs. Additionally, the results of the RNA‐seq analyses showed that a cluster of genes related to the EGFR signalling pathway (such as *Egfr*, *Rho*, *Pnt*, *Sox21a and Ets21C*) was expressed at significantly lower levels in the *ClC*‐*c*‐depleted midguts than in the control midguts (Figure 5A and Figure S4A,B). Among these genes, *Pnt*, *Sox21a* and *Ets21C* have been reported to function downstream of the EGFR signalling pathway and regulate ISC proliferation.^39^, ^40^, ^41^

To confirm these RNA‐seq results, RT‐qPCR analyses were performed. The RT‐qPCR results showed similar expression patterns to those observed in the RNA‐seq data (Figure 5D–G). These findings strongly suggest that *ClC*‐*c* regulates ISC proliferation through the EGFR signalling pathway.

### 3.6 *ClC*‐*c* regulates ISC proliferation through the EGFR signalling

**FIGURE 6:**
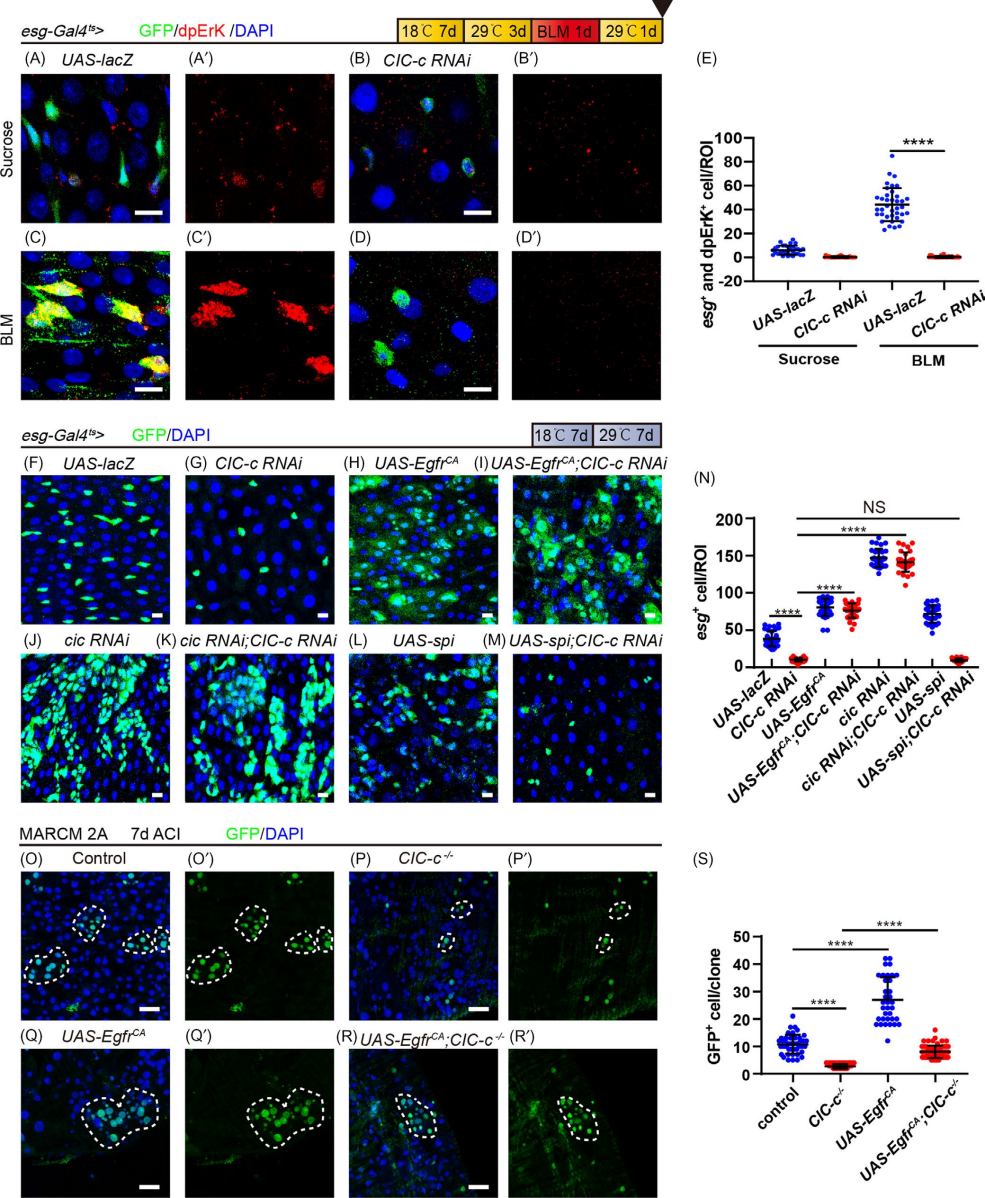
*ClC*‐*c* regulates the proliferation of intestinal stem cells through the EGFR signalling pathway. (A–D) Immunofluorescence images of the R5 region of the midgut of the control flies (A, *esg*‐*Gal4^ts^*‐driven *UAS*‐*lacZ*) with sucrose treatment, *ClC*‐*c*‐depleted flies (B, *esg*‐*Gal4^ts^*‐driven *ClC*‐*c RNAi*) with sucrose treatment, control flies (C, *esg*‐*Gal4^ts^*‐driven *UAS*‐*lacZ*) with BLM treatment, or *ClC*‐*c*‐depleted flies (D, *esg*‐*Gal4^ts^*‐driven *ClC*‐*c RNAi*) with BLM treatment, stained for dpErk. (E) Quantification of the number of the dpErk ^+^ cells in A–D. ROI: 3 × 10^4^ µm² area from the R5 region of the *Drosophila* midgut. Gut *n* ≥ 10; cell *n* ≥ 200. (F–M) Immunofluorescence images from the R5 region of the midgut of the control (F, *esg*‐*Gal4^ts^*‐driven *UAS*‐*lacZ*), *ClC*‐*c*–depleted (G, *esg*‐*Gal4^ts^*‐driven *ClC*‐*c RNAi*), *Egfr^CA^*‐overexpressing (H, *esg*‐*Gal4^ts^*‐driven *UAS*‐*Egfr^CA^*), *Egfr^CA^*‐overexpressing *ClC*‐*c*‐depleted (I, *esg*‐*Gal4^ts^*‐driven *ClC*‐*c RNAi* with *UAS*‐*Egfr^CA^*), *cic*‐depleted (J, *esg*‐*Gal4^ts^*‐driven *cic RNAi*), [*cic* and *ClC*‐*c*]‐depleted (K, *esg*‐*Gal4^ts^*‐driven *ClC*‐*c RNAi* with *cic RNAi*), *spi*‐overexpressing (L, *esg*‐*Gal4^ts^*‐driven *UAS*‐ *spi*), or *spi*‐overexpressing *ClC*‐*c*‐depleted (M, *esg*‐*Gal4^ts^*‐driven *ClC*‐*c RNAi* with *UAS*‐ *spi*) flies. (N) Quantification of the number of the *esg* ^+^ cells in the midgut in F–M. ROI: 2 × 10^4^ µm² area from the R5 region of the *Drosophila* midgut. Gut *n* ≥ 10; ROI *n* ≥ 30. (O–R) Immunofluorescence analyses of the control (O), *ClC*‐*c^d1^*‐null (P), *Egfr*‐overexpressing (Q), and *Egfr*‐overexpressing *ClC*‐*c* null (R) MARCM clones (green) in the midgut 7 days after clonal induction (ACI). (S) Quantification of the cell number per MARCM clone in (M–P). Each dot corresponds to one clone. Gut *n* ≥ 10; clone *n* ≥ 30. ROI, region of interest; BLM, Bleomycin; MARCM, mosaic analysis with a repressible cell marker. DAPI‐stained nuclei are shown in blue. Scale bar represents 10 μm in (A–D, F–M) and 25 μm in (O–R). Error bars represent SDs. Student's *t*‐test, ****p < 0.0001. NS, non‐significant (*p* > 0.05)

Previous studies have shown that the EGFR signalling pathway is vital in ISC proliferation during homeostasis or stress‐induced regeneration.^10^, ^42^ The expression pattern of the EGFR effector mitogen‐activated protein kinase (MAPK) in the midgut was examined by staining the midgut for di‐phosphorylated Erk (dpErk), the active form of MAPK. Signals of phospho‐Erk were detected in the midguts of the control flies after 24 h of BLM treatment (Figure 6A,C,E), but the *ClC*‐*c*‐depleted group showed an unchanged level of Erk activity in response to BLM treatment (Figure 6B,D,E). Moreover, forced constitutive expression of the EGFR receptor (*UAS*‐*Egfr^CA^*) in ISCs was sufficient to induce hyperproliferation along the whole gut epithelium (Figure 6F,H,N). The ISC proliferation defects observed in the *ClC*‐*c*‐depleted flies were significantly restored by overexpressing *Egfr^CA^* (Figure 6F,G,I,N). Consistently, the overexpression of *Egfr* ^CA^ partially rescued the clonal growth inhibition caused by knocking out *ClC*‐*c* (Figure 6O–S). Moreover, activation of the EGFR signalling by knocking down *cic* (an inhibitor of the EGFR signalling pathway) suppressed the cell loss induced by *ClC*‐*c* depletion (Figure 6F,G,J,K,N). These data indicate that *ClC*‐*c* regulates ISC proliferation through the EGFR signalling pathway. Notably, we found that overexpression of the secretory EGFR ligand spi and Krn could not rescue the cell loss induced by the knockdown of *ClC*‐*c* (Figure 6L–N and Figure S5A‐E). These results indicate that knocking down *ClC*‐*c* might inhibit the transduction of EGFR signal from extracellular to intracellular, thereby inhibiting the activation of the EGFR signalling pathway.

### 3.7 Depletion of *ClC*‐*c* inhibits the maturation of Rab5‐labelled early endosomes and Rab7‐labelled late endosomes

**FIGURE 7:**
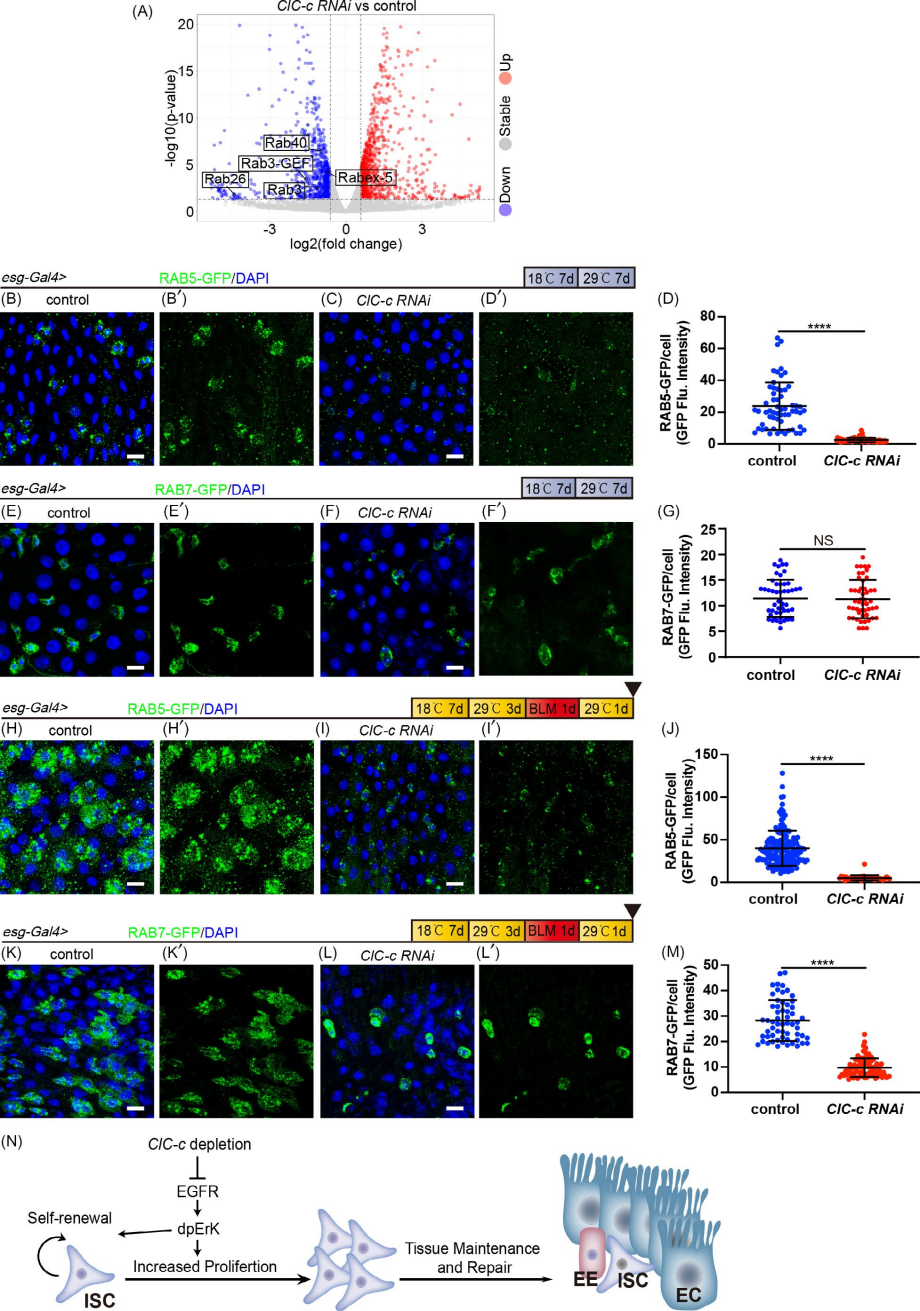
Knocking down *ClC*‐*c* inhibits the maturation of Rab5‐labelled early endosomes and Rab7‐labelled late endosomes. (A) Volcano plots of differentially expressed genes between the *ClC*‐*c*‐depleted *Drosophila* (*esg*‐*Gal4*‐driven *ClC*‐*c RNAi*) midguts and control *Drosophila* (*esg*‐*Gal4*‐driven *UAS*‐*lacZ*) midguts. (B and C) Immunofluorescence images from the R5 region of the midgut of the control (B, *esg*‐*Gal4*‐driven *UAS*‐*RAB5*‐*GFP*) or *ClC*‐*c*‐depleted flies (C, *esg*‐*Gal4*‐driven *UAS*‐*RAB5*‐*GFP*; *ClC*‐*c RNAi*). (D) Quantification of the RAB5‐GFP intensities in (B) and (C). Each dot corresponds to one cell. Gut *n* ≥ 5; cell *n* ≥ 50. (E and F) Immunofluorescence images from the R5 region of the midgut of the control (E, *esg*‐*Gal4*‐driven *UAS*‐*RAB7*‐*GFP*) or *ClC*‐*c*‐depleted (F, *esg*‐*Gal4*‐driven *UAS*‐*RAB7*‐*GFP*; *ClC*‐*c RNAi*) flies. (G) Quantification of the RAB7‐GFP intensities in (E) and (F). Each dot corresponds to one cell. Gut *n* ≥ 5; cell *n* ≥ 50. (H and I) Immunofluorescence images from the R5 region of the midgut of the control (H, *esg*‐*Gal4*‐driven *UAS*‐*RAB5*‐*GFP*) or *ClC*‐*c*‐depleted (I, *esg*‐*Gal4*‐driven *UAS*‐*RAB5*‐*GFP*; *ClC*‐*c RNAi*) flies with BLM treatment. (J) Quantification of the RAB5‐GFP intensities in (H) and (I). Each dot corresponds to one cell. Gut *n* ≥ 10; cell *n* ≥ 50. (K and L) Immunofluorescence images from the R5 region of the midgut of the control (K, *esg*‐*Gal4*‐driven *UAS*‐*RAB7*‐*GFP*) or *ClC*‐*c*‐depleted (L, *esg*‐*Gal4*‐driven *UAS*‐*RAB7*‐*GFP*; *ClC*‐*c RNAi*) flies with BLM treatment. (M) Quantification of the RAB7‐GFP intensities in (K) and (L). Each dot corresponds to one cell. Gut *n* ≥ 10; cell *n* ≥ 50. (N) A model presenting the role of ClC‐c in the control of ISC proliferation. BLM, Bleomycin. DAPI‐stained nuclei are shown in blue. Scale bar represents 10 μm. Error bars represent SDs. Student's *t*‐test, ****p < 0.0001. NS, non‐significant (*p* > 0.05)

Our results indicated that *ClC*‐*c* regulates the proliferation of ISCs through the EGFR signalling; however, how it affects this signalling pathway is unclear. Many studies have demonstrated that endocytosis affects the EGFR signalling. Ligand stimulation causes EGFR to internalize and be transported through the endocytic pathway. Therefore, endocytosis not only regulates the rate of EGFR degradation and circulation but also regulates the EGFR‐mediated signal transduction.^43^, ^44^ By analysing our RNA‐seq data, we found that knocking down *ClC*‐*c* downregulates several endocytosis‐related genes, including *Rab3*, *Rabex*‐*5*, *Rab3*‐*GEF*, *Rab26* and *Rab40* (Figure 7A). These findings strongly suggest that *ClC*‐*c* might affect the vesicle transport system mediated by RAB family proteins. To test this hypothesis, RAB5‐labelled early endosomes and RAB7‐labelled late endosomes were visualized in *esg* ^+^ cells. Under the homeostatic conditions, the RAB5‐GFP signal was remarkably lower in *ClC*‐*c*‐*depleted* group than in the control (Figure 7B–D). There was no difference in the level of the RAB7‐GFP signal after knocking down *ClC*‐*c*, compared with the control level (Figure 7E–G). These results indicate that *ClC*‐*c* depletion affects the activity of early endosomes instead of late endosomes under homeostatic conditions. BLM treatment for 24 h considerably increased the RAB5‐GFP signal compared with the level in the untreated group (Figure 7B,D,H,J). However, knocking down *ClC*‐*c* prevented this increase (Figure 7I,J). Similar results were observed regarding the RAB7‐GFP signal after 24 h of BLM treatment (Figure 7K‐M). These results indicate that knocking down *ClC*‐*c* might inhibit endocytosis.

## 4 DISCUSSION

ClC‐3 is an intracellular ClC protein that functions as a Cl^−^/H^+^ exchanger. Reduced levels of ClC‐3 have been detected in patients with inflammatory bowel disease and mice subjected to DSS‐induced colitis.^34^ *ClC*‐*3* knockout mice are more sensitive to DSS‐induced colitis and show no signs of recovery after treatment.^34^ However, how ClC‐3 regulates intestinal function remains unclear. In this study, we showed in the midgut of adult *Drosophila* that *ClC*‐*c*, the *Drosophila* ortholog of ClC‐3, is only expressed in the ISCs and a small subset of newly generated EBs but not in mature EBs that are about to differentiate into ECs. *ClC*‐*c* was not detected in terminally differentiated ECs or EEs either. Therefore, the expression of *ClC*‐*c* in the midgut of *Drosophila* is stem cell‐specific, suggesting that the regulatory effect of ClC‐3 on intestinal diseases may be related to autonomous regulation of ISC function.

Many studies have shown that ClC‐3 is involved in the regulation of the cell cycle in many cancers.^45^ A recent study has shown that, in DU145 prostate cancer cells, ClC‐3 also acts as a signalling molecule that directly interacts with the stem cell factor SOX2, and then, the two co‐regulate the cell cycle.^46^ Regulation of the cell cycle is very important for stem cell self‐renewal. However, it is still unknown whether ClC‐3 is involved in the regulation of adult stem cells. In this study, we showed that *ClC*‐*c* is involved in the regulation of the ISCs in adult *Drosophila*. Loss of *ClC*‐*c* in the *Drosophila* midgut ISCs leads to their loss and inhibits their proliferation, hindering *Drosophila* midgut ISCs from initiating regeneration through proliferation after an intestinal injury. Our study shows that *ClC*‐*c* also plays an important role in the maintenance of ISC stemness.

The EGFR signalling pathway plays an important role in regulating the renewal and homeostasis of ISCs and is the most important signalling pathway in regulating the proliferation of midgut ISCs in *Drosophila*.^16^, ^47^ Under physiological conditions, both ISC division and the stability of the midgut environment require the activity of the EGFR signalling pathway. Additionally, both compensatory ISC proliferation and midgut regeneration after an intestinal injury require the activity of this pathway.^15^, ^16^, ^39^, ^48^, ^49^, ^50^ However, the upstream of the EGFR signalling in regulatory networks is not fully understood. This study showed that *ClC*‐*c* regulates ISC proliferation by regulating the EGFR signalling pathway. Knocking down *ClC*‐*c* in ISCs inhibited the activation of this pathway, and the stem‐cell loss and proliferation inhibition caused by *ClC*‐*c* deficiency were rescued by activating this pathway. Many studies have proved that endocytosis mediates the onset of EGFR signal attenuation; however, the regulation of endocytosis on EGFR signal is very complex, not just the inhibition of EGFR signal. Studies have shown that the modulation of endosome fusion, reflected by the changes in endosome number and size, could change the distribution of p‐EGFR between endosomes and the EGFR signalling activity of a cell could be regulated by changes in the fusion rate of endosomes. A mild reduction of homotypic early endosome fusion is sufficient to modify cell fate and stop cancer cells proliferating and force them to differentiate instead.^51^ Activated cell‐surface EGFRs are internalized and sorted at the early endosome. The fate of the receptor has important consequences for biological cell outputs, with the recycling pathway favouring cell proliferation. Stress‐activated receptors might also be recycled, thereby promoting cell survival and/or proliferation.^52^ Early endosome damage results in the disruption of EGFR trafficking and compromised EGF‐mediated signalling and survival.^53^ Therefore, inhibition of the EGFR recycling pathway by endocytosis repressor may inhibit the activity of EGFR signalling pathway. EGF‐induced nuclear translocation of EGFR is regulated by early endosomes. The nuclear EGFR can function as a co‐transcription factor for pro‐growth and survival genes. It can also increase PCNA stability and ultimately enhance cellular proliferation.^54^ Although a large number of studies have proved that late endosomes mainly regulate EGFR degradation, some research have also shown that late endosomes have positive regulatory effects on the activity of the EGFR signalling pathway. The p14‐MP1‐MEK1 complex, which is located in the late endosomes, controls endosomal ERK activation and provides the spatial and temporal resolution of ERK signalling that is required to promote proliferation in vivo.^55^ In addition, endocytic also affects signalling pathways that interact with EGFR signalling pathway, such as the JAK/STAT signalling pathway. Injury‐induced JAK/STAT signalling promotes cell proliferation by activating EGFR signalling. Previous studies have shown that blocking trafficking in distinct endosomal compartments leads to an inhibition of the JAK/STAT pathway,^56^ and the internalization and endocytic trafficking of activated Dome allows for compartmentalized signalling to regulate subsets of *Drosophila* JAK/STAT transcriptional targets.^57^ These results strongly supports that the internalization and trafficking are both required for JAK/STAT activity. Therefore, endocytosis may also affect the EGFR signalling pathway through indirect means. In summary, different stages of endocytosis inhibition can inhibit EGFR signalling activity in different ways. Endocytosis is involved in the transduction of the EGFR signal, and our experimental results showed that *ClC*‐*c* deficiency inhibited endocytosis. These results suggest that *ClC*‐*c* deficiency may suppress the EGFR signalling by inhibiting endocytosis. How *ClC*‐*c* deficiency inhibits endocytosis is still unclear, and thus, further research is needed.

In conclusion, this study revealed that *ClC*‐*c* regulates the proliferation of *Drosophila* midgut ISCs by inducing the EGFR signalling pathway (Figure 7N), thereby providing an important basis for further exploration of the regulatory role of *ClC*‐*c* in adult stem cells.

## CONFLICT OF INTERESTS

The authors declare no competing interests.

## AUTHOR CONTRIBUTIONS

JH and HC initiated the project and designed the research; JH, XS, ZZ, DX and KW performed the experiments, data collection and analyses. JH and XS prepared the Figures under the supervision of GW and HC. JH and XS wrote the manuscript. GW and HC proofread and gave advice. All the authors read and approved the final manuscript.

## Supporting information

Figure S1

Figure S2

Figure S3

Figure S4

Figure S5

Tables S1‐S3

## Data Availability

All the RNA‐seq data of this study have been deposited in the Sequence Read Archive (SRA) under BioProject ID PRJNA748595. The authors declare that all other data supporting the findings of this study are available within the article and its Supplementary Information (Figures [Link], [Link], [Link], [Link], [Link], Tables S1‐S3) or from the corresponding author on reasonable request.
