# Supplementary figures and images for: ClC‐c regulates the proliferation of intestinal stem cells via the EGFR signalling pathway in Drosophila
Source: Cell Prolif. 2021 Dec 24;55(1):e13173. doi: 10.1111/cpr.13173 (PMC8780901; doi:10.1111/cpr.13173)

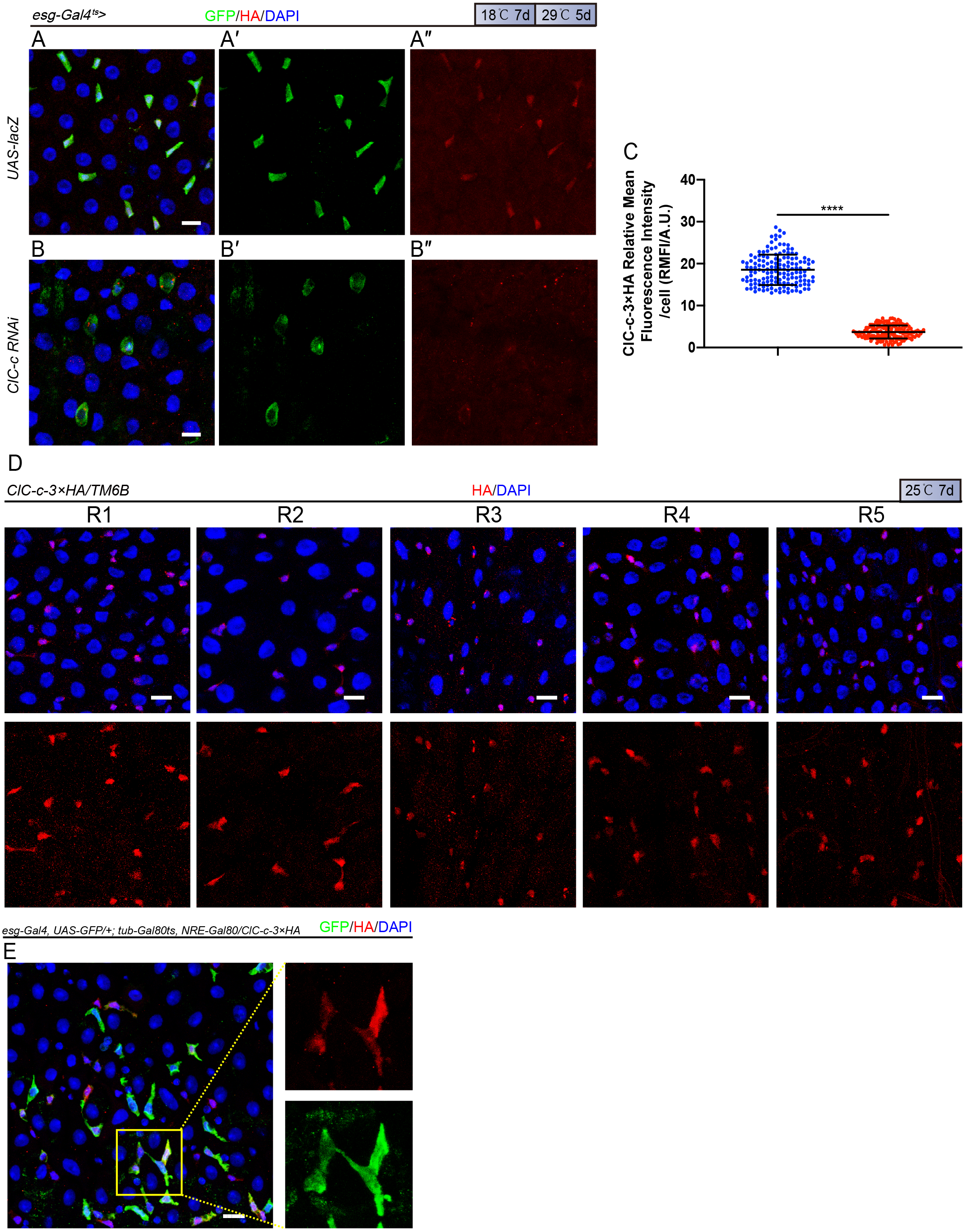

Supplement: Supplementary file 1 — Figure S1 [file CPR-55-e13173-s005.tif]

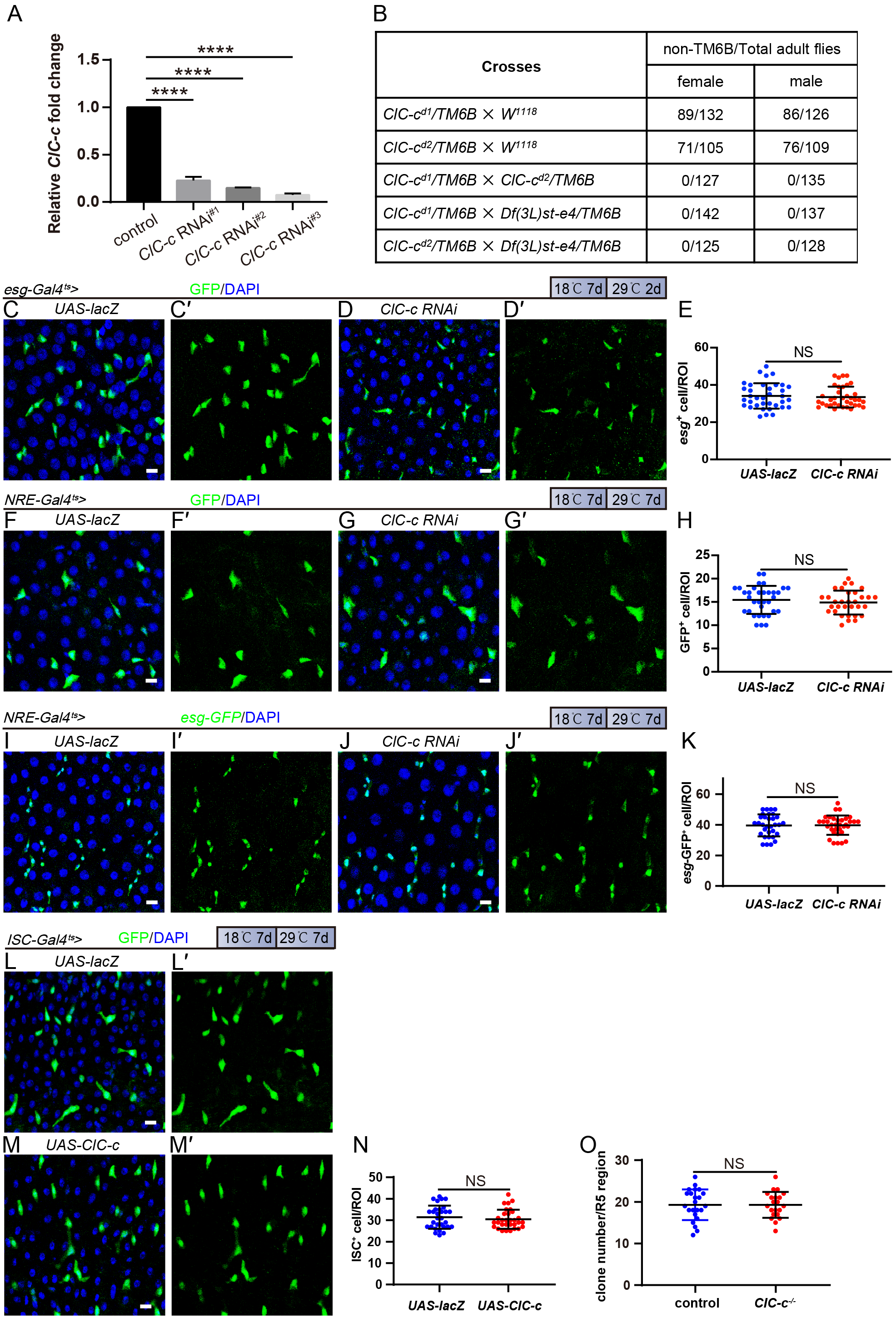

Supplement: Supplementary file 2 — Figure S2 [file CPR-55-e13173-s006.tif]

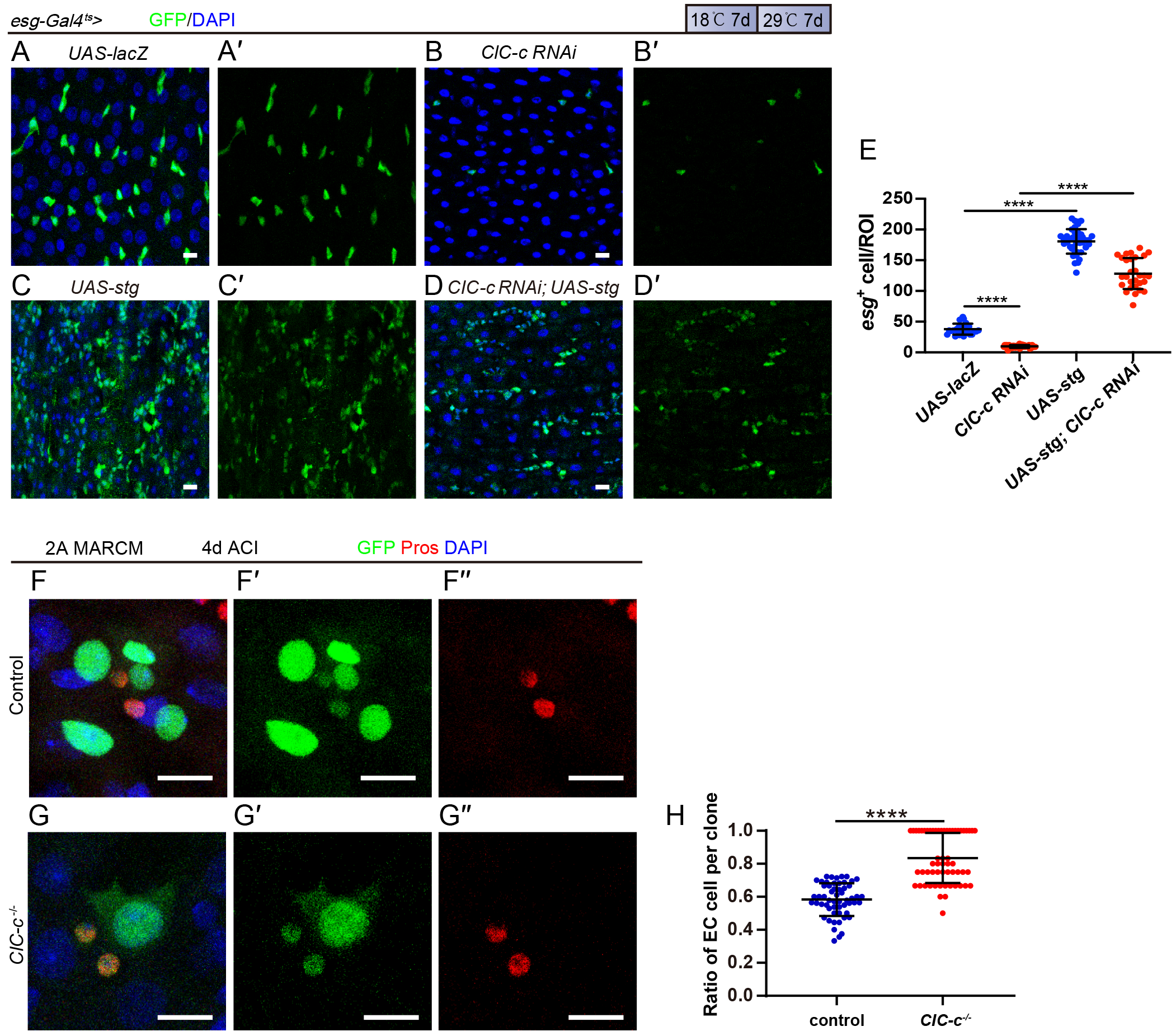

Supplement: Supplementary file 3 — Figure S3 [file CPR-55-e13173-s004.tif]

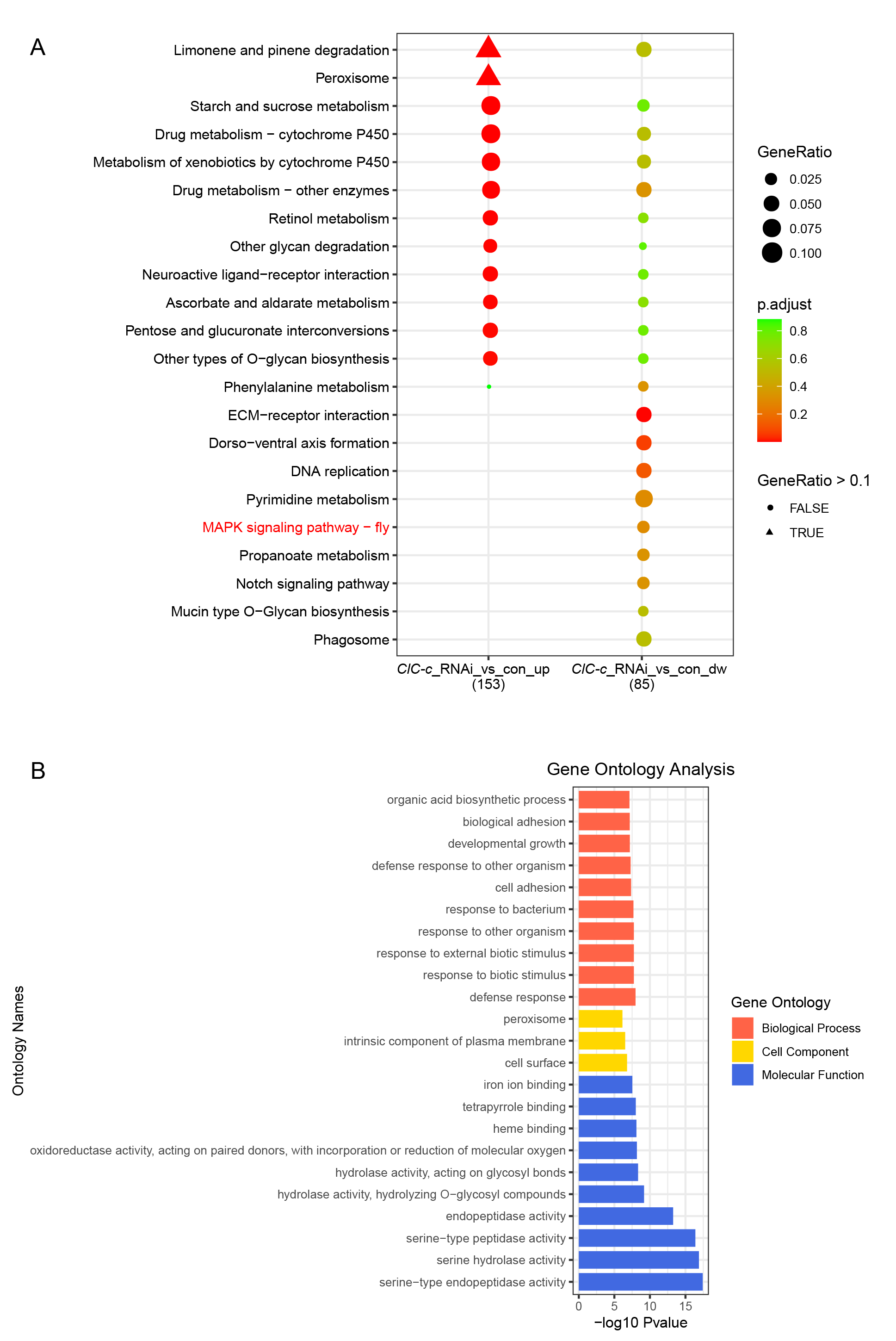

Supplement: Supplementary file 4 — Figure S4 [file CPR-55-e13173-s001.tif]

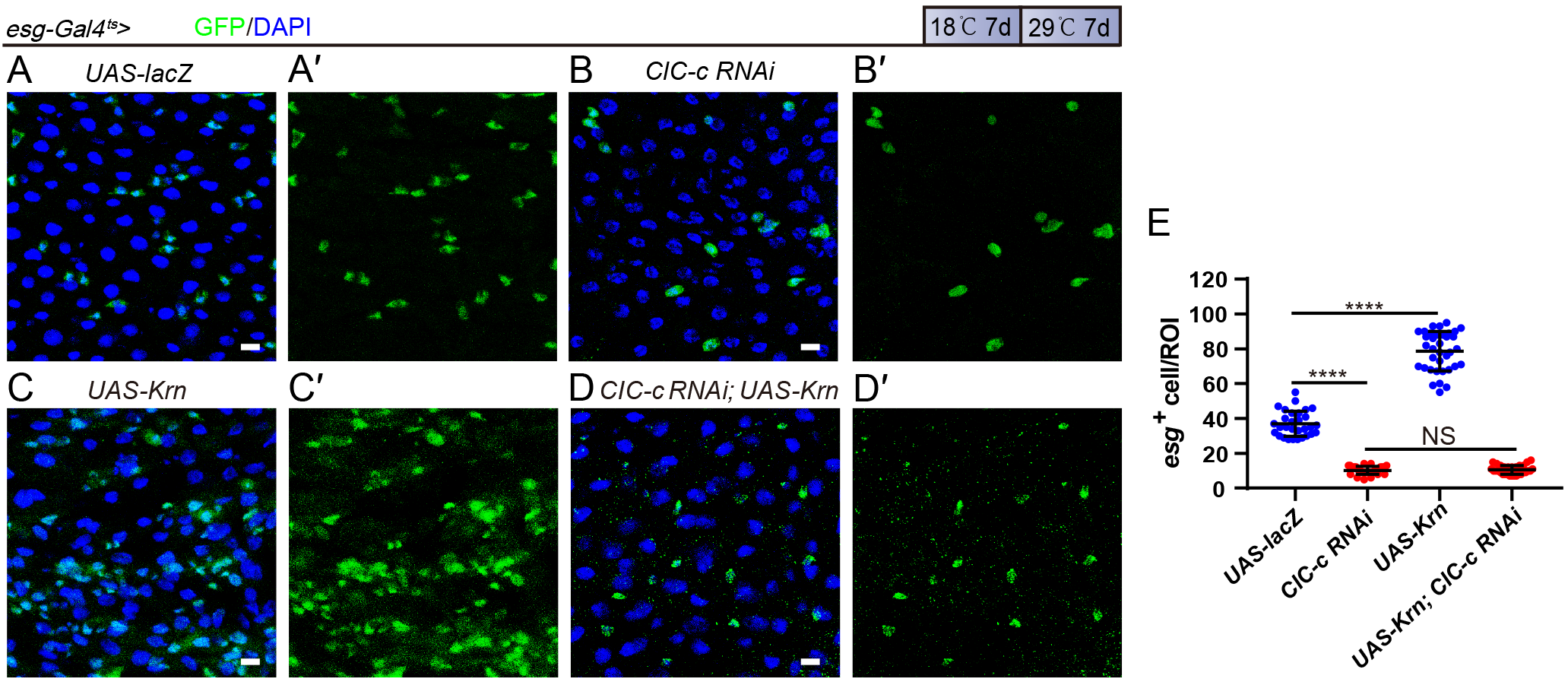

Supplement: Supplementary file 5 — Figure S5 [file CPR-55-e13173-s003.tif]
