# Supplementary material for: ClC‐c regulates the proliferation of intestinal stem cells via the EGFR signalling pathway in Drosophila
Source: Cell Prolif. 2021 Dec 24;55(1):e13173. doi: 10.1111/cpr.13173 (PMC8780901; doi:10.1111/cpr.13173)
Supplement: Supplementary file 6 — Tables S1‐S3 [file CPR-55-e13173-s002.docx]

| **Supplementary Table S1. Full *Drosophila* genotypes as they appear in each figure panel, related to Figures 1-7, and S1-S5** | |
| --- | --- |
| **Figure 1** | |
| **C** | *w^-^; esg-Gal4, UAS-GFP, tub-Gal80^ts^/+; ClC-c-3*×*HA/+* |
| **D** | *w^-^; ; ClC-c-3×HA/TM6B* |
| **E,F** | *w^-^; NRE-Gal4, UAS-GFP, tub-Gal80^ts^/+; ClC-c-3×HA/+* |
| **G** | *w^-^; ; ClC-c-3×HA/TM6B* |
| **H** | *w^-^; Myo1A-Gal4, tub-Gal80^ts^, UAS-GFP/+; ClC-c-3×HA/+* |
| **Figure 2** | |
| **A** | *w^-^; esg-Gal4, UAS-GFP, tub-Gal80^ts^/+; UAS-lacZ/+* |
| **B** | *w^-^; esg-Gal4, UAS-GFP, tub-Gal80^ts^/+; UAS-ClC-c RNAi/+* |
| **D** | *w^-^; esg-Gal4, UAS-GFP/+; tub-Gal80^ts^, NRE-Gal80/UAS-lacZ* |
| **E** | *w^-^; esg-Gal4, UAS-GFP/+; tub-Gal80^ts^, NRE-Gal80/UAS-ClC-c RNAi* |
| **H,I,J,K** | *yw, hsFLP, tub-Gal4, UAS-GFP/+; +/+; tub-Gal80, FRT2A/FRT2A* |
| **L,M,N,O** | *yw, hsFLP, tub-Gal4, UAS-GFP/+; +/+; tub-Gal80, FRT2A/FRT2A, ClC-c^-/-^* |
| **Figure 3** | |
| **A,B,C,D** | *w^-^; ; ClC-c-3×HA/TM6B* |
| **F,G,H,I** | *w^-^; esg-Gal4, UAS-GFP, tub-Gal80^ts^/+; UAS-lacZ/+* |
| **J,K,L,M** | *w^-^; esg-Gal4, UAS-GFP, tub-Gal80^ts^/+; UAS-ClC-c RNAi/+* |
| **Figure 4** | |
| **A,D,F,K** | *w^-^; esg-Gal4, UAS-GFP, tub-Gal80^ts^/+; UAS-lacZ/+* |
| **B,E,G,L** | *w^-^; esg-Gal4, UAS-GFP, tub-Gal80^ts^/+; UAS-ClC-c RNAi/+* |
| **C** | *w^-^; esg-Gal4, UAS-GFP, tub-Gal80^ts^/+; UAS-rpr/+* |
| **N** | *yw, hsFLP, tub-Gal4, UAS-GFP/+; +/+; tub-Gal80, FRT2A/FRT2A* |
| **O** | *yw, hsFLP, tub-Gal4, UAS-GFP/+; +/+; tub-Gal80, FRT2A/FRT2A, ClC-c^-/-^* |
| **P** | *yw, hsFLP, tub-Gal4, UAS-GFP/+; UAS-stg /+; tub-Gal80, FRT2A/FRT2A* |
| **Q** | *yw, hsFLP, tub-Gal4, UAS-GFP/+; UAS-stg /+; tub-Gal80, FRT2A/FRT2A, ClC-c^-/-^* |
| **Figure 6** | |
| **A,C,F** | *w^-^; esg-Gal4, UAS-GFP, tub-Gal80^ts^/+; UAS-lacZ/+* |
| **B,D,G** | *w^-^; esg-Gal4, UAS-GFP, tub-Gal80^ts^/+; UAS-ClC-c RNAi/+* |
| **H** | *w^-^; esg-Gal4, UAS-GFP, tub-Gal80^ts^/+; UAS-Egfr^CA^/+* |
| **I** | *w^-^; esg-Gal4, UAS-GFP, tub-Gal80^ts^/+; UAS-ClC-c RNAi/UAS-Egfr^CA^* |
| **J** | *w^-^; esg-Gal4, UAS-GFP, tub-Gal80^ts^/UAS-cic RNAi; +/+* |
| **K** | *w^-^; esg-Gal4, UAS-GFP, tub-Gal80^ts^/UAS-cic RNAi; UAS-ClC-c RNAi/+* |
| **L** | *w^-^; esg-Gal4, UAS-GFP, tub-Gal80^ts^/+; UAS-spi/+* |
| **M** | *w^-^; esg-Gal4, UAS-GFP, tub-Gal80^ts^/+; UAS-spi/UAS-ClC-c RNAi* |
| **O** | *yw, hsFLP, tub-Gal4, UAS-GFP/+; +/+; tub-Gal80, FRT2A/FRT2A* |
| **P** | *yw, hsFLP, tub-Gal4, UAS-GFP/+; +/+; tub-Gal80, FRT2A/FRT2A, ClC-c^-/-^* |
| **Q** | *yw, hsFLP, tub-Gal4, UAS-GFP/+; +/+; tub-Gal80, FRT2A/FRT2A, UAS-Egfr^CA^* |
| **R** | *yw, hsFLP, tub-Gal4, UAS-GFP/+; +/+; tub-Gal80, FRT2A/FRT2A, ClC-c^-/-^, UAS-Egfr^CA^* |
| **Figure 7** | |
| **B,H** | *w^-^; esg-Gal4/+; UAS-Rab5-GFP/tub-Gal80^ts^* |
| **C,I** | *w^-^; esg-Gal4/+; UAS-Rab5-GFP/tub-Gal80^ts^, UAS-ClC-c RNAi* |
| **E,K** | *w^-^; esg-Gal4/UAS-Rab7-GFP; tub-Gal80^ts^/+* |
| **F,L** | *w^-^; esg-Gal4/UAS-Rab7-GFP; tub-Gal80^ts^/UAS-ClC-c RNAi* |
| **Figure S1** | |
| **A** | *w^-^; esg-Gal4, UAS-GFP, tub-Gal80^ts^/+; UAS-lacZ/+* |
| **B** | *w^-^; esg-Gal4, UAS-GFP, tub-Gal80ts/+; UAS-ClC-c RNAi/+* |
| **D** | *w^-^; ; ClC-c-3×HA/TM6B* |
| **E** | *w^-^; esg-Gal4, UAS-GFP/+; tub-Gal80^ts^, NRE-Gal80/ClC-c-3×HA* |
| **Figure S2** | |
| **C** | *w^-^; esg-Gal4, UAS-GFP, tub-Gal80^ts^/+; UAS-lacZ/+* |
| **D** | *w^-^; esg-Gal4, UAS-GFP, tub-Gal80^ts^/+; UAS-ClC-c RNAi/+* |
| **F** | *w^-^; NRE-Gal4, UAS-GFP, tub-Gal80^ts^/+; UAS-lacZ/+* |
| **G** | *w^-^; NRE-Gal4, UAS-GFP, tub-Gal80^ts^/+; UAS-ClC-c RNAi/+* |
| **I** | *w^-^; NRE-Gal4, UAS-GFP, tub-Gal80^ts^/ esg-GFP; +/+* |
| **J** | *w^-^; NRE-Gal4, UAS-GFP, tub-Gal80^ts^/ esg-GFP; UAS-ClC-c RNAi/+* |
| **L** | *w^-^; esg-Gal4, UAS-GFP/+; tub-Gal80^ts^, NRE-Gal80/UAS-lacZ* |
| **M** | *w^-^; esg-Gal4, UAS-GFP/+; tub-Gal80^ts^, NRE-Gal80/UAS-ClC-c* |
| **Figure S3** | |
| **A** | *w^-^; esg-Gal4, UAS-GFP, tub-Gal80^ts^/+; UAS-lacZ/+* |
| **B** | *w^-^; esg-Gal4, UAS-GFP, tub-Gal80^ts^/+; UAS-ClC-c RNAi/+* |
| **C** | *w^-^; esg-Gal4, UAS-GFP, tub-Gal80^ts^/+; UAS-stg/+* |
| **D** | *w^-^; esg-Gal4, UAS-GFP, tub-Gal80^ts^/+; UAS-stg/UAS-ClC-c RNAi* |
| **F** | *yw, hsFLP, tub-Gal4, UAS-GFP/+; +/+; tub-Gal80, FRT2A/FRT2A* |
| **G** | *yw, hsFLP, tub-Gal4, UAS-GFP/+; +/+; tub-Gal80, FRT2A/FRT2A, ClC-c^-/-^* |
| **Figure S5** | |
| **A** | *w^-^; esg-Gal4, UAS-GFP, tub-Gal80^ts^/+; UAS-lacZ/+* |
| **B** | *w^-^; esg-Gal4, UAS-GFP, tub-Gal80^ts^/+; UAS-ClC-c RNAi/+* |
| **C** | *w^-^; esg-Gal4, UAS-GFP, tub-Gal80^ts^/+; UAS-krn/+* |
| **D** | *w^-^; esg-Gal4, UAS-GFP, tub-Gal80^ts^/+; UAS-krn/UAS-ClC-c RNAi* |
|  |  |
|  |  |

| **Supplementary Table S2 (related to Materials and Methods). Regent Table** | | |
| --- | --- | --- |
| **Reagent** | **Source** | **Dilution** |
| Chicken polyclonal anti-GFP | Abcam Cat# ab13970 RRID:AB_300798 | 1: 1000 |
| Rabbit Polyclonal anti-GFP | Proteintech Cat# 50430-2-AP RRID:AB_11042881 | 1: 1000 |
| Rabbit anti-HA (C29F4) | Cell Signaling Technology Cat# 3724 RRID:AB_1549585 | 1: 1000 |
| Mouse anti-Delta | DSHB Cat# C594.9B RRID:AB_528194 | 1: 100 |
| Mouse anti-Prospero | DSHB Cat# MR1A RRID:AB_528440 | 1: 200 |
| Rabbit anti-phosphoHistone H3 (Ser10) | Millipore Cat# 06-570 RRID:AB_310177 | 1: 1000 |
| Mouse anti-pMAPK (Erk1/2) (137F5) | Cell Signaling Technology Cat# 4695 RRID: AB_390779 | 1: 200 |
| Alexa 488 | Invitrogen | 1: 1000 |
| Alexa 568 | Invitrogen | 1: 1000 |
| Alexa 647 | Invitrogen | 1: 1000 |

| **Supplementary Table S3. Primer sequences used in this study.** | |
| --- | --- |
| Target1-*ClC-c*-sgRNA-F | GTTTCCGCTGAAGGGCAGCA |
| Target1-*ClC-c*-sgRNA-R | TGCTGCCCTTCAGCGGAAAC |
| Target2-*ClC-c*-sgRNA F | AGTCCAGGCGATGTGATCGG |
| Target2-*ClC-c*-sgRNA R | CCGATCACATCGCCTGGACT |
| gRNA1 for knock-in F | GGATAACGAAGATCCCAATA |
| gRNA1 for knock-in R | TATTGGGATCTTCGTTATCC |
| gRNA2 for knock-in F | GCGCTCGCCCAACTTCTGAT |
| gRNA2 for knock-in R | ATCAGAAGTTGGGCGAGCGC |
| *ClC-c*-F | CGGAACTTTTCGTGACCATC |
| *ClC-c*-R | TGGACAAATGCCATGCTTTA |
| *Egfr*-F | TTATCCGTGGAGGAGGAGAAGTA |
| *Egfr*-R | AGGTGGCACCATAGGCATACTTT |
| *pnt*-F | ACGCCCTATGATGCTCAATC |
| *pnt*-R | TATCCAGACCCAAGGTGCTC |
| *Ets21C*-F | CCGGGCACTCAGGTACTACT |
| *Ets21C*-R | CATACTGGAGGCCGGATCT |
| *Sox21a*-F | GCCGAGTGGAAATTACTCACCGAA |
| *Sox21a*-R | TGCGACGTGGTCGATACTTGTAGT |
| *Delta*-F | AGGCTTGTACTGCAACCAGGATCT |
| *Delta*-R | TGAGCACTTTCTCCTCGCACATCT |
| *esg*-F | GCCGCAGGATTTGTGCGTAAAGAA |
| *esg*-R | ATGACCCTGCTGATTGATGGTCCT |
| *Rp49*-F | TCGATATGCTAAGCTGTC |
| *Rp49*-R | GGCATCAGATACTGTCCCTTG |
